# Differential transcriptional profiles identify microglial- and macrophage-specific gene markers expressed during virus-induced neuroinflammation

**DOI:** 10.1186/s12974-019-1545-x

**Published:** 2019-07-20

**Authors:** Ana Beatriz DePaula-Silva, Carlos Gorbea, Daniel J. Doty, Jane E. Libbey, John Michael S. Sanchez, Tyler J. Hanak, Demián Cazalla, Robert S. Fujinami

**Affiliations:** 1 Department of Pathology, University of Utah School of Medicine, 15 North Medical Drive East, 2600 EEJMRB, Salt Lake City, UT 84112 USA; 2 Department of Biochemistry, University of Utah, 15 North Medical Drive East, 4100 EEJMRB, Salt Lake City, UT 84112 USA

**Keywords:** Microglia, Macrophages, Cell-specific markers, RNA-Seq, Neuroinflammation, Immune response, Cell surface markers, CNS, TMEV, Viral-induced neuroinflammation

## Abstract

**Background:**

In the healthy central nervous system (CNS), microglia are found in a homeostatic state and peripheral macrophages are absent from the brain. Microglia play key roles in maintaining CNS homeostasis and acting as first responders to infection and inflammation, and peripheral macrophages infiltrate the CNS during neuroinflammation. Due to their distinct origins and functions, discrimination between these cell populations is essential to the comprehension of neuroinflammatory disorders. Studies comparing the gene profiles of microglia and peripheral macrophages, or macrophages in vitro-derived from bone marrow, under non-infectious conditions of the CNS, have revealed valuable microglial-specific genes. However, studies comparing gene profiles between CNS-infiltrating macrophages and microglia, when both are isolated from the CNS during viral-induced neuroinflammation, are lacking.

**Methods:**

We isolated, via flow cytometry, microglia and infiltrating macrophages from the brains of Theiler’s murine encephalomyelitis virus-infected C57BL/6 J mice and used RNA-Seq, followed by validation with qPCR, to examine the differential transcriptional profiles of these cells. We utilized primary literature defining subcellular localization to determine whether or not particular proteins extracted from the transcriptional profiles were expressed at the cell surface. The surface expression and cellular specificity of triggering receptor expressed on myeloid cells 1 (TREM-1) protein were examined via flow cytometry. We also examined the immune response gene profile within the transcriptional profiles of these isolated microglia and infiltrating macrophages.

**Results:**

We have identified and validated new microglial- and macrophage-specific genes, encoding cell surface proteins, expressed at the peak of neuroinflammation. TREM-1 protein was confirmed to be expressed by infiltrating macrophages, not microglia, at the peak of neuroinflammation. We also identified both unique and redundant immune functions, through examination of the immune response gene profiles, of microglia and infiltrating macrophages during neurotropic viral infection.

**Conclusions:**

The differential expression of cell surface-specific genes during neuroinflammation can potentially be used to discriminate between microglia and macrophages as well as provide a resource that can be further utilized to target and manipulate specific cell responses during neuroinflammation.

**Electronic supplementary material:**

The online version of this article (10.1186/s12974-019-1545-x) contains supplementary material, which is available to authorized users.

## Background

In the brain, microglial cells are resident cells of the parenchyma, comprising 5–20% of the non-neuronal glial cells [1, 2]. Microglial cells arise from erythro-myeloid precursors in the yolk sac and, during early stages of embryonic development, migrate to the central nervous system (CNS) [3, 4]. Known as resident macrophages of the CNS, microglial cells perform a multitude of important functions during homeostasis and disease. In healthy brains, microglia are found in their ramified morphology, functioning as sentinels by sampling their surroundings and maintaining homeostasis through the clearance of cellular debris [5–8]. In this state, microglia can be referred to as homeostatic microglia [9]. Additionally, microglia can shift from the homeostatic to the reactive state in response to insults that lead to inflammation, such as pathogen infection of the CNS, and in response to damage-associated molecular patterns. Reactive microglia upregulate major histocompatibility complex class-II (MHC-II), become phagocytic, secrete proinflammatory cytokines, and can function as antigen-presenting cells [9–12].

In addition to microglia, other myeloid cells such as perivascular macrophages and meningeal macrophages can be found at the boundaries between the CNS and the periphery [13, 14]. Moreover, peripheral macrophages can infiltrate into the CNS parenchyma upon the breakdown of the blood-brain barrier as a result of CNS damage and inflammation [15, 16]. Different from microglia, macrophages are derived from monocytes, which originate from bone marrow hematopoietic stem cells (reviewed in [17]). Like microglia, macrophages possess high plasticity, adapting and changing phenotype depending on the surrounding environment, and they contribute to proinflammatory responses as well as healing responses [18, 19].

As components of the innate immune response, microglia and macrophages are frequently associated with CNS pathologies such as neuroinflammation. The presence of reactive microglia and infiltration of macrophages into the CNS are often observed during brain inflammation (encephalitis), Parkinson’s disease, multiple sclerosis, traumatic brain injury, and brain tumorigenesis, among others [15, 20–25]. Upon activation, macrophages can acquire distinct phenotypes such as the classically defined M1, or proinflammatory, and M2, or anti-inflammatory, polarization states. While M1 polarization correlates with the induction of inflammation through the secretion of proinflammatory cytokines such as interleukin (IL)-6 and tumor necrosis factor (TNF)-α, induction of the M2 phenotype results in secretion of anti-inflammatory mediators, favoring tissue repair and homeostasis [1, 5, 18, 19]. It is important to mention that change in macrophage phenotype is a spectrum and not a binary state, implying that both states can be found simultaneously. Similarly, microglia can also acquire distinct phenotypes as they transition from the homeostatic state to a more reactive state, as discussed earlier [9–12].

Due to microglia and macrophages performing unique functions in the CNS during inflammation, discrimination between these two cell populations is essential. For several years, CD11b and Iba1 (ionized calcium-binding adapter molecule 1) cell surface expression molecules were used as markers to identify these myeloid cells in the CNS; however, these markers are expressed by both macrophages and microglia, and cannot be used to discriminate between these two cell types [26]. Although specific microglial markers such as P2Y purinoceptor 12 (P2ry12) and transmembrane protein 119 (TMEM119) have recently been identified by comparing gene expression profiles between microglia and circulating monocytes [27, 28], more recent work has demonstrated that TMEM119 is also expressed in macrophages in the lymph nodes [29], while P2ry12 expression is decreased during microglial activation [27, 30], suggesting that these markers might not be as specific for or constitutively expressed on microglia as first thought.

Using a multiplex assay and infection with the JHM strain of mouse hepatitis virus (JHMV), Savarin et al. [31] recently demonstrated that bone marrow-derived macrophages and microglia have distinct gene expression profiles during JHMV-induced demyelination. Although this method is quantitative and provided valuable information, this approach is limited in the types of genes that can be measured. Furthermore, comparison of gene expression profiles, using RNA sequencing (RNA-Seq), between microglia and infiltrating macrophages, when both cell types are isolated from the CNS during viral-induced neuroinflammation, has not yet been reported. In this study, we sought to identify cell surface markers that are uniquely expressed by infiltrating macrophages and microglia during virus-induced brain inflammation and also to determine functional differences and similarities between these two cell populations in the context of the immune response. Although several groups have used genetic and pharmacological approaches to study microglia, these methods have resulted in contradictory findings since these techniques were shown to non-specifically target other myeloid cells, such as monocytes and macrophages [32, 33].

In order to study microglial and infiltrating macrophage populations present in the CNS during encephalitis, we used our mouse model of viral-induced seizures/epilepsy [34–36]. In this model, intracerebral (i.c.) infection of C57BL/6 J mice with Theiler’s murine encephalomyelitis virus (TMEV) results in mice experiencing acute seizures that start on day 3, peak at day 6–7, and resolve by day 10 post infection. These acute seizures are characterized by CNS infiltration of immune cells such as macrophages and lymphocytes [15, 34–36]. We have previously determined that macrophage infiltration, more specifically IL-6-producing macrophages [15, 22, 35, 36], but not T cell or neutrophil infiltration, is important for seizure development [22]. Recently, we and others have also demonstrated that macrophages and microglia have unique roles in controlling viral infection in the brain [37–39].

Using this mouse model, we isolated infiltrating macrophages and microglia from the brains of TMEV-infected and phosphate-buffered saline (PBS)-injected mice and, through RNA-Seq, we identified new signature genes that are differentially expressed by microglia and infiltrating macrophages during neuroinflammation. Our results (a) identified and validated genes that are uniquely expressed at the cell surface of microglia or macrophages, which can potentially be used to discriminate between these two cell populations; and (b) demonstrated that microglia and macrophages have both redundant and unique immune response functions during neuroinflammation.

## Methods

### Animal experiments

Four-week-old, male C57BL/6 J mice were obtained from the Jackson Laboratory (Bar Harbor, ME). All animal experiments were reviewed and approved by the University of Utah Institutional Animal Care and Use Committee (Protocols #15-08004 and #18-06006) and conducted in accordance with the guidelines prepared by the Committee on Care and Use of Laboratory Animals, Institute of Laboratory Animals Resources, National Research Council. Animals were maintained at a maximum of five mice per cage on a 12 h light/12 h dark cycle at 22 °C. Food and water were available ad libitum*.* Mice were maintained on Teklad sterilizable rodent diet (autoclaved diet) (cat. #8656; Harlan Laboratories, Indianapolis, IN). All efforts were made to minimize suffering. Mice were euthanized through an overdose of isoflurane.

### TMEV infection

C57BL/6 J mice were anesthetized with isoflurane by inhalation and infected i.c. with 3 × 10^5^ plaque-forming units (PFU) (unless stated otherwise) of the Daniels (DA) strain of TMEV at a final volume of 20 μl per mouse. Mice were mock-infected with 20 μl PBS as a control. The DA strain of TMEV was propagated as previously described [40].

### Flow cytometry

Mice were euthanized on the indicated days and perfused with PBS. Brains and spleens were obtained and placed in tubes containing PBS and kept on ice. Cells were mechanically isolated and suspended in RPMI-1640 media (Mediatech, Herndon, VA) supplemented with 1% l-glutamine (Mediatech), 1% antibiotics (Mediatech), 50 μM 2-mercaptoethanol (Sigma, St. Louis, MO), and 10% Cosmic calf serum (CCS) (Hyclone, Logan, UT). Briefly, mouse brains were finely sliced using a razor blade and placed in a 15 ml Falcon tube with digestion solution [1 mg/ml of collagenase D (Sigma), 0.1 mg/ml DNase I (Roche, Basel, Switzerland)], and incubated for 20 min at 37 °C. Digestion solution with cells was transferred to a petri dish and the cell suspension was homogenized by continuous pipetting. Cells were then transferred to a 15 ml Falcon tube with 8 ml of RPMI-1640 media supplemented with 10% CCS. Cells were washed and isolated by centrifugation (30 min, 700×*g* at 4 °C) using a continuous 37% Percoll gradient (Sigma). Cells from spleens were mechanically isolated and further purified by incubating in ACK red blood cell lysis buffer (0.15 M NH_4_Cl, 10 mM KHCO_3_, pH 7.3). Isolated cells were washed, counted using an automated cell counter (TC-20, BioRad, Hercules, CA), and treated with Fc blocker (Biolegend, San Diego, CA). Next, cells were immunostained with the indicated anti-mouse antibodies for 30 min at 4 °C [rat anti-mouse CD45 V500 (BD Bioscience, San Jose, CA), rat anti-mouse CD45-phycoerythrin (PE) (BD Bioscience), CD11b-allophycocyanin (APC) (eBioscience, San Diego, CA), mouse triggering receptor expressed on myeloid cells 1 (TREM-1)-PE (R&D Systems, Minneapolis, MN), CD3 Brilliant Violet (BV) 421 (Biolegend), MHC-II-PE (Biolegend), MHC-II BV 421 (Biolegend)]. Stained cells were then analyzed by flow cytometry. Brain-derived cells were stained and analyzed individually for each mouse. Gating was determined by fluorescence-minus-one (FMO) with isotype-matched immunoglobulin control. More specifically, FMO controls contained each antibody conjugate used in the experiment except one, with the addition of the appropriate isotype control for the excluded fluorochrome. This was performed for each fluorochrome using TMEV-infected brain samples. Live cells were determined by forward and side scatter fluorescence on a BD LSRFortessa X-20 Cell Analyzer (BD Bioscience). Flow cytometry data analysis was performed using FlowJo software (Tree Star, Inc., Ashland, OR).

### RNA isolation and library preparation for RNA-Seq

Brains were harvested and cells were isolated as described above. Isolated cells from TMEV-infected mice or PBS-injected mice were pooled together. Cells were FACS-sorted (Aria Cell Sorter, BD Bioscience) and immediately stored in 1 ml of TRIzol (Invitrogen, Carlsbad, CA) at − 80 °C. Cell sorting was performed at the Flow Cytometry Core Facility at the University of Utah. RNA was isolated by phase separation by adding chloroform to samples in TRIzol, followed by centrifugation (12,000×*g*, 15 min at 4 °C). The aqueous phase, which contains the RNA, was transferred to a new RNase/DNase-free tube and RNA was then precipitated with isopropanol and glycoBlue (Invitrogen). Oligo d(T)25 magnetic beads (New England Biolabs, Ipswich, MA) were used to enrich for polyadenylated (polyA) mRNA [41]. Briefly, purified total RNA was suspended in water and heated for 5 min at 65 °C. One volume of 2× binding buffer (40 mM Tris-HCl, pH 7.5, 1  M NaCl, 1% SDS, 2 mM EDTA, 10 mM DTT) was added to each sample and the mix was transferred to an Eppendorf tube containing oligo d(T)25 magnetic beads. Samples were incubated in a thermomixer for 30 min at 25 °C. We next washed the beads twice using wash buffer 1 (20  mM Tris-HCl, pH 7.5, 500 mM NaCl, 0.1% SDS, 1  mM EDTA, 5 mM DTT) followed by two washes with wash buffer 2 (20 mM Tris-HCl, pH 7.5, 500 mM NaCl, 1 mM EDTA). Beads were incubated with low salt buffer (20 mM Tris-HCl, pH 7.5, 200 mM NaCl, 1 mM EDTA) and polyA RNA was eluted with elution buffer (20 mM Tris-HCl, pH 7.5, 1 mM EDTA). The SMARTer Stranded RNA-Seq Kit (Takara, Mountain View, CA) was used to prepare cDNA libraries for RNA-Seq according to the manufacturer’s protocol. HiSeq 50 cycle single-read sequencing was performed on an Illumina HiSeq 2500 instrument. RNA-Seq and the bioinformatic analyses of the results obtained from the RNA-Seq experiment were done by the High Throughput Genomics Core Facility in the Huntsman Cancer Institute at the University of Utah.

### Reverse transcription and quantitative PCR

TMEV-infected mice and PBS-injected mice were euthanized and perfused with PBS. Brains and spleens were harvested and cells isolated as described above. Cells were FACS-sorted and RNA was isolated using an RNeasy Mini Kit (Qiagen, Chatsworth, CA). A DNase digestion was performed on the column during the RNA purification, as per the manufacturer’s recommendations. The RNA was quantified with a Qubit Fluorometer (Life Technologies, Carlsbad, CA) and stored at − 80 °C until use. cDNA was synthesized using 100 ng of RNA and the High Capacity cDNA Reverse Transcription Kit with MultiScribe Reverse Transcriptase (Applied Biosystems, Waltham, MA), in a 20 μl reaction volume. The quantitative PCR (qPCR) amplification reaction was performed in a 12 μl final volume reaction containing 6 μl of PowerUp SYBR green 2× Master Mix (Applied Biosystems), 1.5 μl of primers (0.5 μM) (Additional file 2: Table S1), 2 μl of diluted cDNA, and 2.5 μl of water. The amplification reaction was performed in a QuantStudio 6 (Applied Biosystems) LightCycler, using the following parameters: 50 °C for 2 min, 95 °C for 10 min, 1 cycle; 95 °C for 15 s, 55 °C for 15 s, and 72 °C for 1 min, 50 cycles, followed by a melting curve that was generated by heating from 60 to 95 °C with a ramp rate of 0.05 °C per second, with continuous measurement of the fluorescence. Reactions were performed in duplicate in at least two independent experiments. Relative expression levels were determined by the standard curve method using β-actin as an endogenous control for normalization.

### Differential gene expression analysis

The mouse GRCm38 FASTA and GTF files were downloaded from Ensembl release 94 and the reference database was created using STAR version 2.6.1b [42] with splice junctions optimized for 50 base-pair reads. Reads were trimmed of adapters using cutadapt 1.16 [43] and then aligned to the reference database using STAR in two pass mode to output a BAM file sorted by coordinates. Mapped reads were assigned to annotated genes in the GTF file using featureCounts version 1.6.3 [44]. The output files from cutadapt, FastQC, Picard CollectRnaSeqMetrics, STAR, and featureCounts were summarized using MultiQC [45] to check for any sample outliers. Differentially expressed genes were identified using a 5% false discovery rate with DESeq2 version 1.20.0 [46]. Significant genes overlapping gene sets, pathways, and curated studies were analyzed using Illumina’s BaseSpace Correlation Engine (http://www.illumina.com). Identification of significantly enriched pathways in the REACTOME database was conducted using the Gene Set Enrichment Analysis (GSEA) preranked tool [47, 48] (http://www.gsea-msigdb.org/gsea/index.jsp).

### Statistical analysis

Statistical analyses were performed using Prism 5 software (GraphPad, San Diego. CA). The two-tailed, non-paired Student’s *t* test was used for comparison of two groups. Differences were considered to be statistically significant at *p* < 0.05.

## Results

### Isolation and identification of microglial and infiltrating macrophage populations in the mouse brain during viral infection

**Fig. 1:**
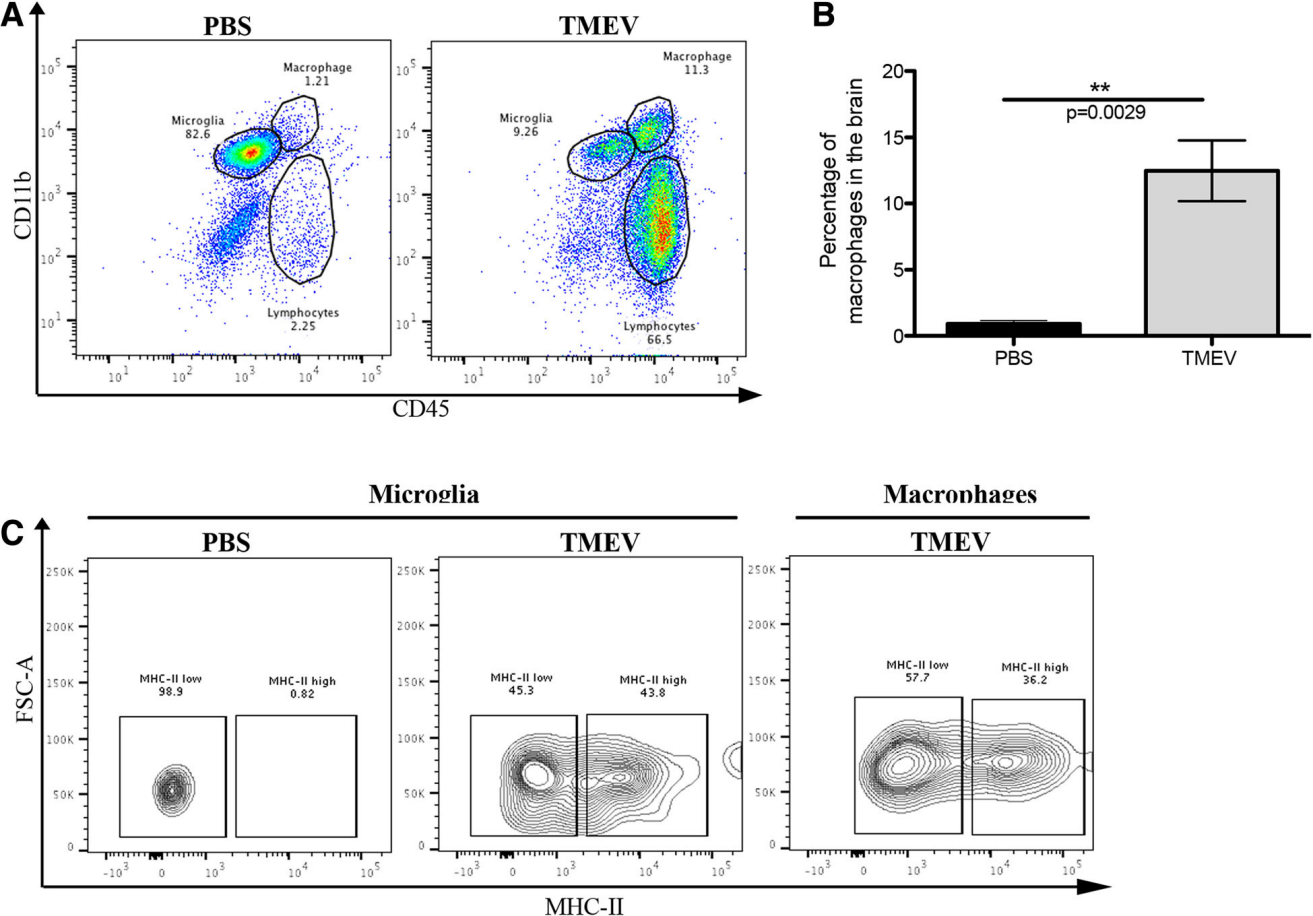
Identification/characterization of microglia and infiltrating macrophages within the brains of TMEV-infected and PBS-injected mice. **a** Representative flow cytometry plots showing microglia (CD45^lo/int^ CD11b^+^), macrophages (CD45^hi^ CD11b^+^), and lymphocytes (CD45^+^ CD11b^lo/int^) isolated from the brains of PBS-injected and TMEV-infected mice at 7 days post infection (d.p.i.). **b** Quantification of macrophage infiltration into the brains of TMEV-infected (*n* = 15) and PBS-injected (*n* = 4) mice at 7 d.p.i. Data represents the mean ± standard error of the mean (SEM). **c** Major histocompatibility complex class-II (MHC-II) expression on microglia and macrophages at 7 d.p.i. Gates were set according to FMO as described in the “Methods” section. Student’s *t* test, **p < 0.01, degrees of freedom (df) = 3

To characterize microglia and infiltrating macrophages in relation to their gene expression profiles during brain inflammation and to determine functional differences between these cell populations, C57BL/6 J mice were i.c. infected with TMEV (total of 30 mice) or PBS-injected (total of 40 mice) and euthanized at 6 days post infection (d.p.i.), the peak of disease. Brains were harvested and cells isolated by Percoll gradient centrifugation. Isolated cells were next stained with antibodies, and microglia and infiltrating macrophages were cell-sorted using flow cytometry based on CD45 and CD11b levels of expression; microglia are defined as CD45^lo/int^ CD11b^+^ and macrophages as CD45^hi^ CD11b^+^ [49]. The characteristic expression of CD11b and CD45 by microglia and infiltrating macrophages is demonstrated in Fig. 1a. As expected, a significantly higher percentage of infiltrating macrophages was found in the brains of TMEV-infected mice compared to PBS-injected mice upon quantification (*p* < 0.01, df = 3, Student’s *t* test) (Fig. 1b).

Analysis of the expression of the MHC-II molecule on microglia and infiltrating macrophages was used to determine the activation status of these two cell populations [15, 50]. While the microglia of PBS-injected mice showed a low level of MHC-II expression at 7 d.p.i., microglia and infiltrating macrophages of TMEV-infected mice expressed a high level of MHC-II at 7 d.p.i., demonstrating that these cells are activated toward an inflammatory phenotype (M1) (Fig. 1c). Based on the expression of the activation marker MHC-II, macrophages and microglia isolated from the brains of TMEV-infected mice are henceforth referred to as infiltrating macrophages and reactive microglia, respectively, whereas microglia isolated from the brains of PBS-injected mice are referred to as homeostatic microglia.

### Reactive microglia and infiltrating macrophages have distinct gene profiles following TMEV infection of the CNS

**Fig. 2:**
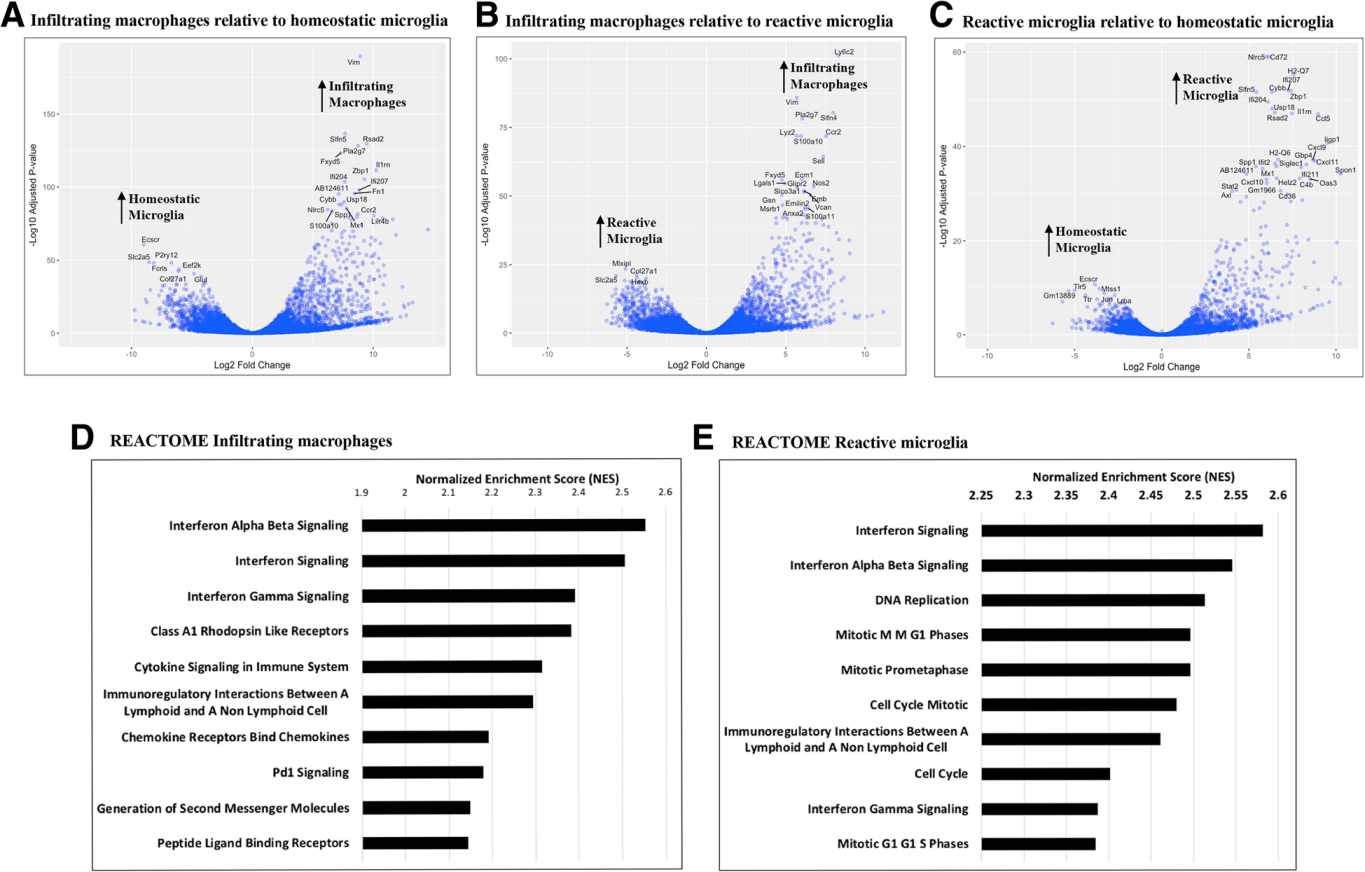
Microglia and infiltrating macrophages in the CNS have distinct gene profiles during neuroinflammation. Volcano plots of differential gene expression profiles showing the magnitude on the *x*-axis (Log2 fold change) and significance on the y-axis (-Log10 adjusted *p* value) for **a** infiltrating macrophages relative to homeostatic microglia, **b** infiltrating macrophages relative to reactive microglia, and **c** reactive microglia relative to homeostatic microglia. Upright arrows indicate set of genes upregulated in microglia or macrophages. Adjusted *p* value < 0.05 and a Log2 fold change > 2 are considered statistically significant. REACTOME pathway analysis for genes upregulated in infiltrating macrophages (**d**) and reactive microglia (**e**), was conducted using GSEA preranked tool

We next compared the transcriptional profiles of infiltrating macrophages, reactive microglia, and homeostatic microglia. RNA was extracted from the sorted cells and enriched for polyA+ mRNA, which was then used to synthesize cDNA libraries that were subjected to RNA-Seq. A principal component analysis (PCA) was conducted (R studio) using normalized Log2 values from the top 500 variable genes. Based on the expression profiles, we found that infiltrating macrophages, reactive microglia, and homeostatic microglia cluster distantly from each other (data not shown), suggesting that these cells have distinct gene expression profiles. Volcano plots illustrating genes that are highly and differentially expressed in infiltrating macrophages relative to homeostatic microglia, infiltrating macrophages relative to reactive microglia, and reactive microglia relative to homeostatic microglia are depicted in Fig. 2a–c, respectively.

Comparison of the gene expression profiles between homeostatic microglia and infiltrating macrophages (Fig. 2a), and reactive microglia and infiltrating macrophages (Fig. 2b) revealed that, besides C-C motif chemokine receptor 2 (*Ccr2*), lymphocyte antigen 6 complex, locus C2 (*Ly6c2*), and lysozyme 2 (*Lyz2*), which are genes normally found to be expressed in macrophages, infiltrating macrophages also expressed, among other genes, vimentin (*Vim*), which is a protein secreted by activated macrophages; interferon-activated gene 204 (*Ifi204*), which is the murine ortholog of human *Ifi16* and is induced by interferon; S100 calcium binding protein A10 (*S100a10*), that has been shown to be important for macrophage migration [51]; and methionine sulfoxide reductase (*Msrb1*), which plays a role in actin polymerization, redox metabolism and, recently, has been shown to be important for the induction of anti-inflammatory cytokines by macrophages [52]. Furthermore, we found that canonical microglial signature genes such as *P2ry12*, Fc receptor-like S (*Fcrls*), and solute carrier family 2, facilitated glucose transporter member 5 (*Slc2a5*) were upregulated in homeostatic microglia compared to infiltrating macrophages (Fig. 2a). However, when we compared gene expression profiles of infiltrating macrophages with reactive microglia (Fig. 2b), transcripts of *P2ry12* and *Fcrls* seemed to be expressed at lower levels compared to homeostatic microglia (Fig. 2a), suggesting that microglial activation modulates expression of microglial signature genes.

To determine representative pathways that are significantly induced by differentially expressed genes, we conducted GSEA [47, 48] using the REACTOME database. The ten most significantly enriched pathways for infiltrating macrophages and reactive microglia are depicted in Fig. 2d, e, respectively. Whereas several distinct pathways were found to be enriched either in infiltrating macrophages or reactive microglia, interferon signaling was upregulated in both of these cell populations. Class A1 rhodopsin-like receptors, which are the largest group of G protein-coupled receptors (GPCRs), cytokine, and chemokine signaling were also enriched in infiltrating macrophages (Fig. 2d). Interestingly, we found cell cycle-related genes to be highly enriched in reactive microglia (Fig. 2e) but not in infiltrating macrophages (Fig. 2d). This agrees with the fact that upon activation, microglia undergo biochemical changes that allow these cells to rapidly proliferate. Furthermore, activation of microglia and macrophages allows these cells to initiate an immune response, which includes the production of chemokines and cytokines that results in further activation of other immune cells of the innate and adaptive immune responses.

**Fig. 3:**
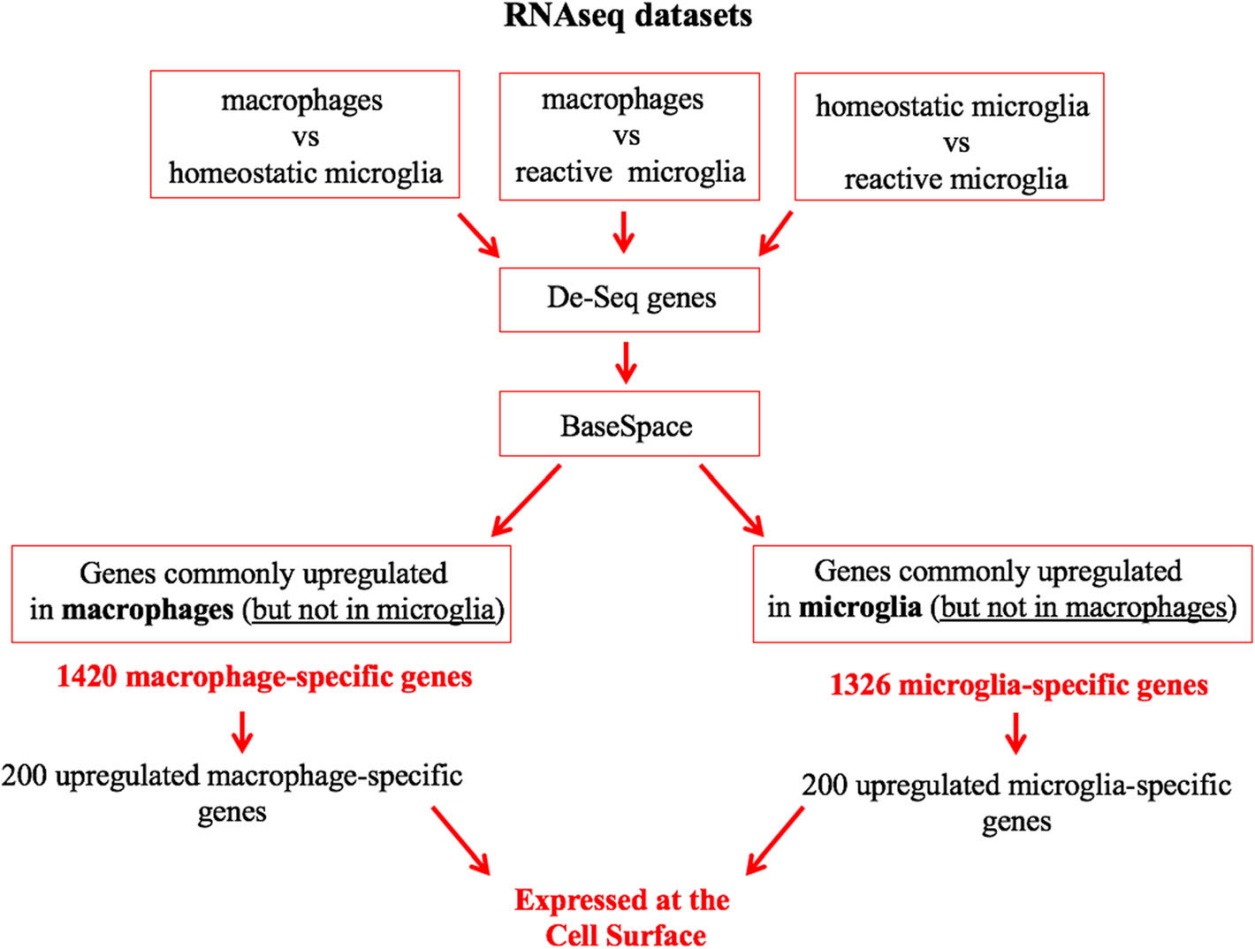
Scheme demonstrating the workflow for samples and different methods used to perform RNA-Seq analysis. Differentially expressed genes determined via RNA-Seq were further analyzed using the BaseSpace correlation engine to identify genes that were uniquely upregulated in infiltrating macrophages (1420 genes) or in microglia (1326 genes). Next, we determined which gene products were expressed at the cell surface

**Fig. 4:**
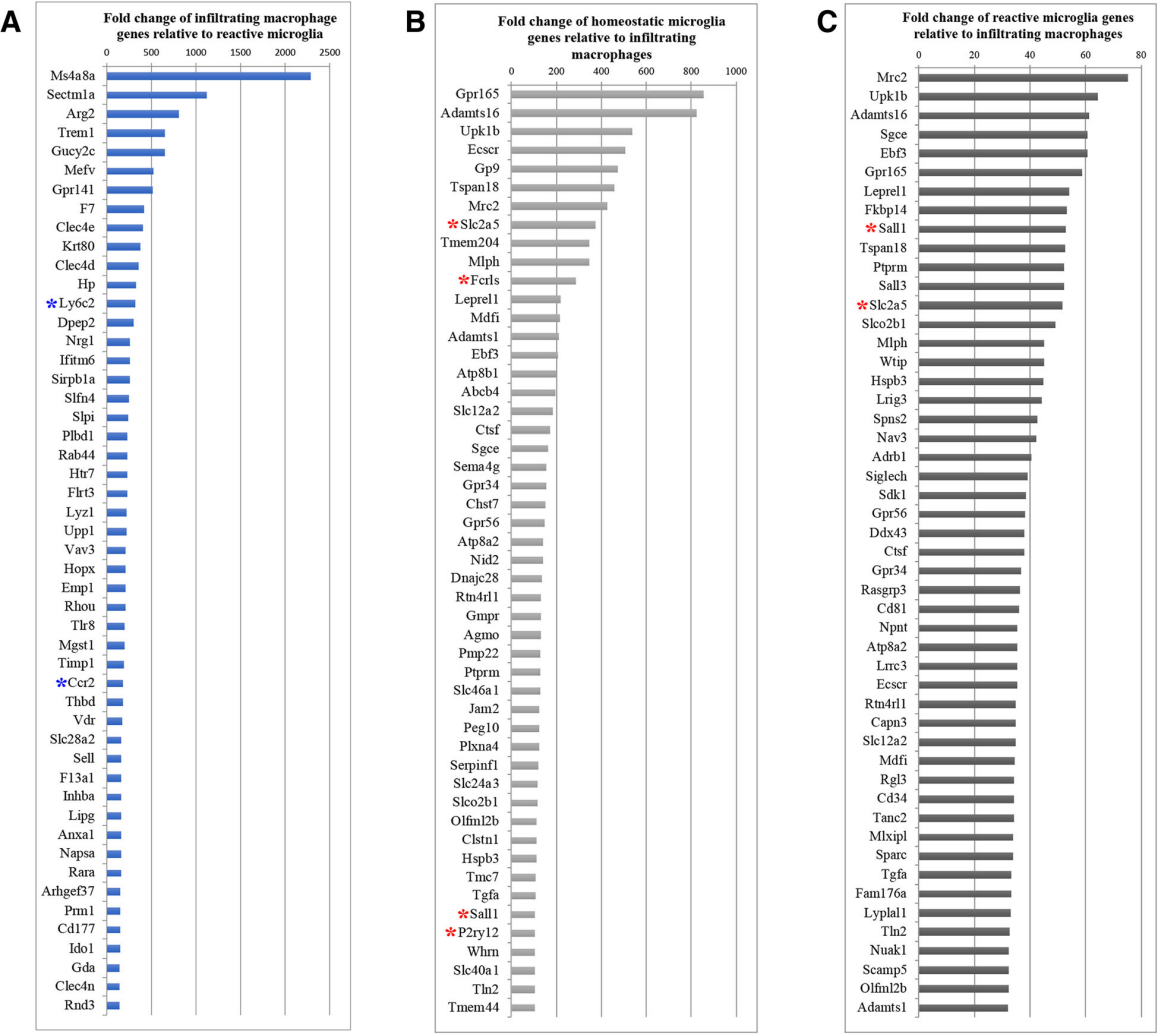
Expression of macrophage- and microglial-specific genes during neuroinflammation. Fold change of the 50 most highly enriched genes in **a** infiltrating macrophages relative to reactive microglia, **b** homeostatic microglia relative to infiltrating macrophages, and **c** reactive microglia relative to infiltrating macrophages. The blue asterisk highlights genes that are usually found in macrophages while the red asterisk highlights genes known to be specifically expressed in microglia. Analysis was performed using the BaseSpace correlation engine

To identify proteins uniquely expressed in infiltrating macrophages or in microglia (homeostatic and reactive), we used the BaseSpace correlation engine (Illumina) and performed analyses on the gene expression profiles generated from our RNA-Seq datasets. A schematic of the workflow for our analyses is shown in Fig. 3. We selected genes that appeared only in infiltrating macrophages or in microglia but were not expressed in both cell populations. A total of 1420 genes were identified to be exclusively expressed in infiltrating macrophages and 1326 genes were found to be uniquely expressed in microglia, suggesting that although these cell populations have many similarities, they are in fact significantly distinct from each other. Finally, we sorted these genes based on the fold change into 50 highly expressed genes specific to infiltrating macrophages (Fig. 4a) or to microglia (Fig. 4b, c).

Among the most highly expressed genes found in infiltrating macrophages were membrane-spanning 4-domains, subfamily A, member 8A (*Ms4a8a*); arginase, type II (*Arg2*); and *Trem-1* (Fig. 4a). Interestingly, TREM-1 protein expression in macrophages is associated with amplification of the inflammatory immune response and secretion of proinflammatory cytokines, such as IL-6 [53]. Additionally, while *Arg2* seems to have a dual role in both M1 and M2 macrophages, expression of *Ms4a8a* is related to the induction of M2 macrophages [54]. Furthermore, we found *Ly6c2* and *Ccr2*, both of which are associated with M1 (inflammatory) macrophages, to be highly expressed in infiltrating macrophages (Fig. 4a, blue asterisk). These results suggest that, at least at 6 d.p.i., there is a heterogeneous population of proinflammatory and anti-inflammatory macrophages in the CNS. Since we found genes associated with M2 macrophages to be highly expressed at this time-point, it suggests that either infiltrating macrophages are acquiring an anti-inflammatory phenotype by altering their polarization, or anti-inflammatory macrophages are infiltrating the CNS.

Among the differentially and highly expressed microglial genes, we found canonical signatures such as *Slc2a5*, *Fcrls*, spalt-like transcription factor 1 (*SalI1*), and *P2ry12* (Fig. 4b, c, red asterisk) [27, 28, 55]. Sialic acid-binding immunoglobulin-type lectin H (*SiglecH*), a specific microglia marker [56] that was suggested to be expressed when these cells are polarized toward the inflammatory phenotype, was also highly expressed by reactive microglia in our assay (Fig. 4c). Therefore, our results widely identified distinct genes that are differentially and specifically expressed by microglia and infiltrating macrophages during neuroinflammation.

### qPCR of selected cell surface markers to distinguish between infiltrating macrophages and microglia in the CNS following viral infection

**Fig. 5:**
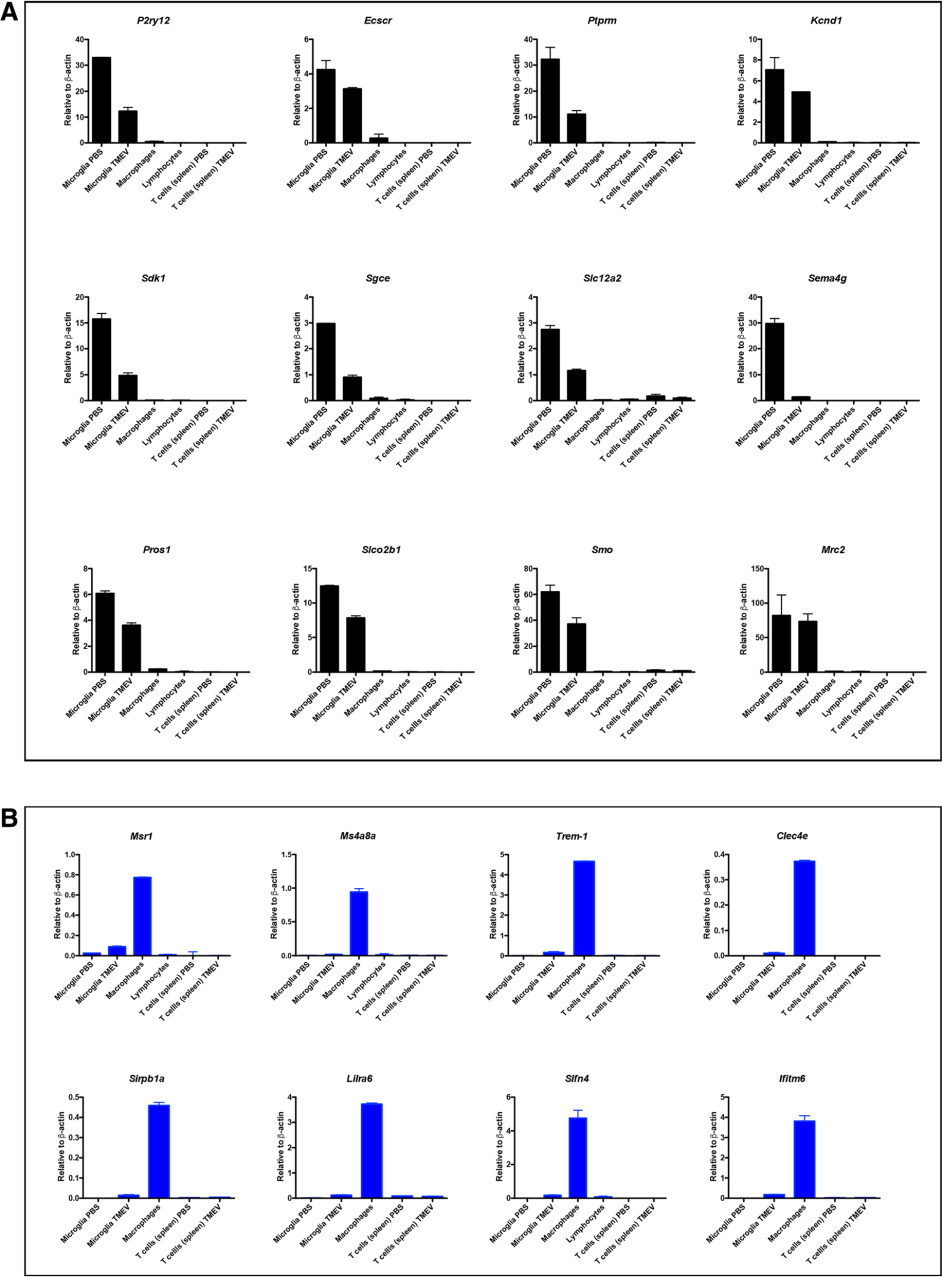
Validation of gene expression of microglial- and macrophage-specific genes. **a** Level of mRNA expression of microglial-specific genes. **b** Level of mRNA expression of macrophage-specific genes. Cells obtained from the brains of TMEV-infected (*n* = 10) and PBS-injected (*n* = 20) mice, and from the spleens of TMEV-infected (*n* = 5) and PBS-injected (*n* = 5) mice were cell-sorted as follows: microglia (CD45^lo/int^ CD11b^+^), macrophages (CD45^hi^ CD11b^+^), brain lymphocytes (CD45^+^ CD11b^lo/int^), and splenic T cells (CD45^+^ CD3^+^). RNA was extracted and quantified by qPCR. Levels were normalized to β-actin. Data are presented as means ± standard deviation. Shown is one of two independent experiments

Since we were interested in identifying unique cell surface markers that could be used to distinguish microglia from infiltrating macrophages in the CNS, we next determined which gene products were possibly expressed at the cell surface (Fig. 3). Although most of the available online databases can predict trans-membrane proteins, the accurate cellular localization (whether the protein is expressed at the cell surface) is not precise. Therefore, we utilized primary literature to define subcellular localization to determine whether or not a particular protein was expressed at the cell surface. We selected a total of 50 gene candidates that were all cell surface molecules of either infiltrating macrophages or microglia (Table 1) and we performed qPCR to further validate our findings. Microglia and infiltrating macrophages were isolated and cell-sorted from the brains of TMEV-infected and PBS-injected mice as described above. We also cell-sorted infiltrating lymphocytes (CD45^+^ CD11b^lo/int^) from the brains and T cells (CD45^+^ CD3^+^) from the spleens of TMEV-infected or PBS-injected mice for use as internal specificity controls, allowing us to determine whether or not selected genes were expressed in microglia or infiltrating macrophages but not expressed in other cell populations. RNA was isolated from each cell population and used to perform qPCR. Notably, in addition to *P2ry12*, we found sidekick cell adhesion molecule 1 (*Sdk1*), endothelial cell surface expressed chemotaxis and apoptosis regulator (*Ecscr*), sarcoglycan epsilon (*Sgce*), solute carrier family 12 member 2 (*Slc12a2*), Semaphorin 4G (*Sema4g*), smoothened homolog (*Smo*), mannose receptor C type 2 (*Mrc2*), protein tyrosine phosphatase, receptor type M (*Ptprm*), potassium voltage-gated channel subfamily D member 1 (*Kcnd1*), protein S (*Pros1*), solute carrier organic anion transporter family member 2B1 (*Slco2b1*), solute carrier family 46 member 1 (*Slc46a1*), junctional adhesion molecule 2 (*Jam2*), and Plexin A4 (*Plxna4*) to be exclusively expressed in microglia (Fig. 5a and Additional file 1: Figure S1A). Notably, with the exception of *P2ry12*, *Mrc2*, and *Pros1*, all the other transcripts have not been previously described or confirmed by qPCR as being expressed in microglia. Intriguingly, all identified genes were downregulated after microglial activation.

**Table 1:** List of 50 cell surface candidate genes specifically expressed by microglia or infiltrating macrophages

Microglia cell surface enriched genes		Macrophage cell surface enriched genes	
Gene symbol	Gene name	Gene symbol	Gene name
*Ecscr*	Endothelial cell surface expressed chemotaxis and apoptosis regulator	*Ecm1*	Extracellular matrix protein 1
*Scamp5*	Secretory carrier membrane protein 5	*Emb*	Embigin
*Ptprm*	Protein tyrosine phosphatase, receptor type M	*Slco3a1*	Solute carrier organic anion transporter Family member 3a1
*Kcnd1*	Potassium voltage-gated channel subfamily D, member 1	*Emp1*	Epithelial membrane protein 1
*Sdk1*	Sidekick cell adhesion molecule 1	*Ms4a7*	Membrane spanning 4-domains A7
*Clstn1*	Calsyntenin-1	*Gpr132*	G protein-coupled receptor 132
*Sgce*	Sarcoglycan epsilon	*Pdpn*	Podoplanin
*Slc12a2*	Solute carrier family 12, member 2	*Alcam*	Activated Leukocyte Cell Adhesion Molecule
*Cadm1*	Cell adhesion molecule 1	*Ms4a6d*	Membrane-spanning 4-domains, subfamily A, member 6a
*Sema4g*	Semaphorin 4G	*Msr1*	Macrophage scavenger receptor-1
*Pros1*	Protein S	*Kcnn4*	Potassium calcium-activated channel subfamily N, member 4
*Slco2b1*	Solute carrier organic anion transporter family, member 2b1	*Scarf1*	Scavenger receptor class F member 1
*Rtn4rl1*	Reticulon 4 Receptor Like 1	*Antxr2*	Anthrax toxin receptor 2
*Cmtm4*	CKLF-like MARVEL transmembrane domain-containing protein 4	*Fam26f*	Calcium homeostasis modulator family, member 6
*Fat3*	Fat atypical cadherin 3	*Ms4a8a*	Membrane-spanning 4-domains subfamily A, member 8A
*Smo*	Smoothened homolog	*Wfdc17*	Activated macrophage/microglia WAP domain protein
*Mrc2*	Mannose receptor C type 2	*Lilr4b*	Leukocyte immunoglobulin-like receptor, subfamily B, member 4B
*Jam2*	Junctional adhesion molecule 2	*Trem-1*	Triggering receptor expressed on myeloid cells 1
*Plxna4*	Plexin A4	*Gpr141*	Probable G-protein coupled receptor 141
*Slc46a1*	Solute carrier family 46, member 1	*Clec4e*	C-type lectin domain family 4, member E
*Agmo*	Alkylglycerol monooxygenase (Tmem195)	*Sirpb1a*	SIRP-beta B-type
*Gpr165*	G protein-coupled receptor 165	*Bst1*	Bone marrow stromal cell antigen 1
*Tmem204*	Transmembrane Protein 204	*Lilra6*	Leukocyte immunoglobulin-like receptor subfamily A member 6
*P2ry12*	P2Y purinoceptor 12	*Slfn4*	Schlafen-4
		*Ifitm6*	Interferon-induced transmembrane protein 6
		*Iqgap2*	IQ motif-containing GTPase activating protein 2

Cell surface localization of microglia- (left) or infiltrating macrophage- (right) specific gene products was determined using primary literature

Furthermore, we found that *Ms4a8a*, Schlafen 4 (*Slfn4*), *Trem-1*, interferon-induced transmembrane protein 6 (*Ifitm6*), leukocyte immunoglobulin-like receptor subfamily A, member 6 (*Lilra6*), SIRP-beta B-type (*Sirpb1a*), and C-type lectin domain family 4, member E (*Clec4e*) (Fig. 5b) were specifically expressed by infiltrating macrophages. Macrophage scavenger receptor 1 (*Msr1*) is predominantly expressed by infiltrating macrophages but a small induction is also observed in reactive microglia (Fig. 5b). Potassium calcium-activated channel subfamily N member 4 (*Kcnn4*) and membrane-spanning 4-domains, subfamily A, member 6A (*Ms4a6d*), among other genes (Additional file 1: Figure S1B), were not uniquely expressed by microglia or infiltrating macrophages. Altogether, our results identified new gene markers that are specifically expressed by microglia or by infiltrating macrophages during viral encephalitis.

### TREM-1 is expressed by CNS-infiltrating macrophages

**Fig. 6:**
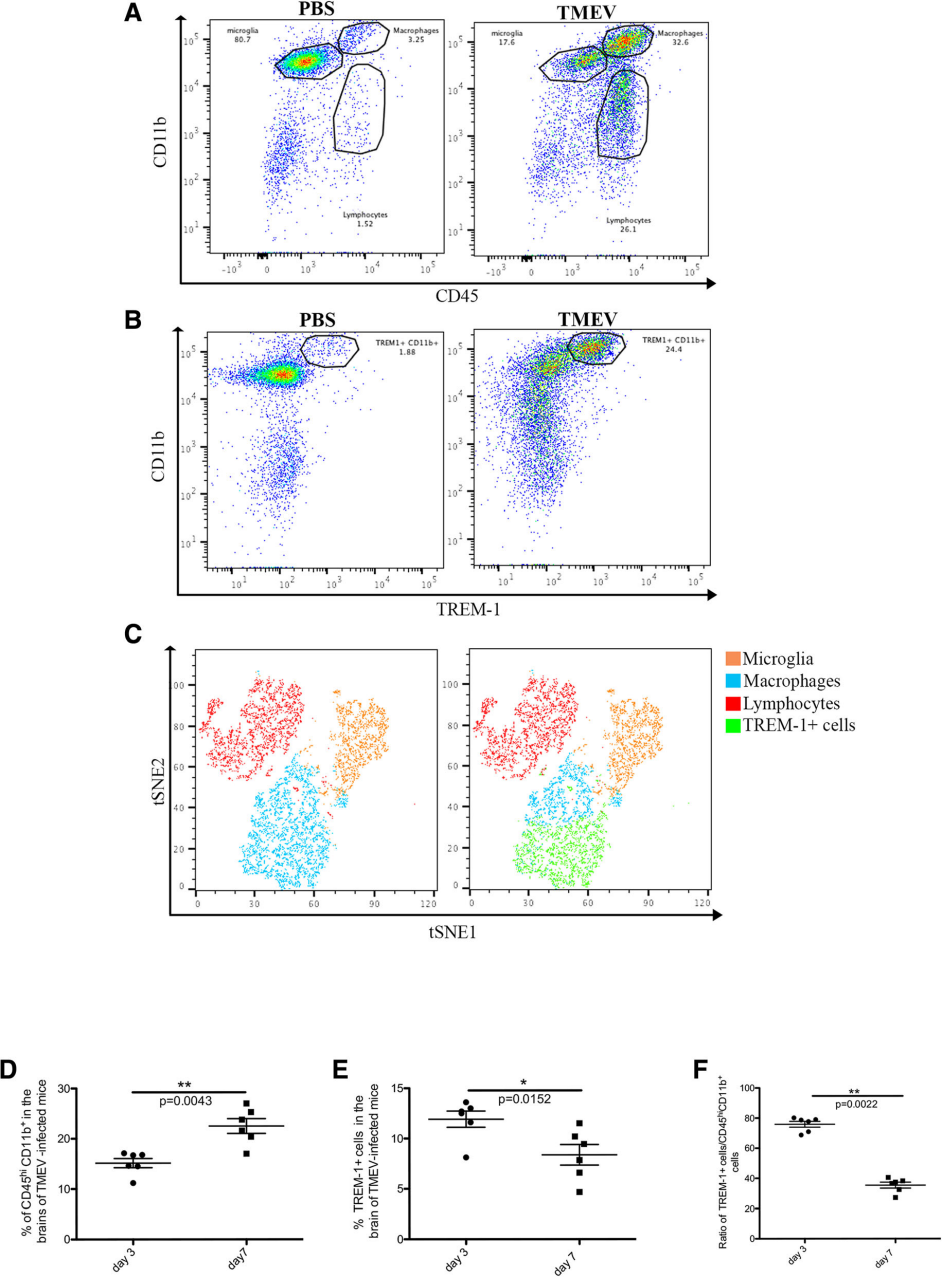
TREM-1 is expressed by infiltrating macrophages. C57BL/6 J mice were intracerebrally infected with TMEV or PBS-injected and mice were euthanized at 3 d.p.i. **a** Cells were isolated and microglia (CD45^lo/int^ CD11b^+^), macrophages (CD45^hi^ CD11b^+^), and lymphocytes (CD45^+^ CD11b^lo/int^) were identified by flow cytometry. **b** Schematic flow plot of TREM-1 expressing cells in PBS-injected and TMEV-infected brains. **c** Overlay of manually gated microglia, macrophages, lymphocytes, and TREM-1-expressing cells, demonstrating TREM-1 is exclusively expressed in infiltrating macrophages. **d** Quantification of macrophages (CD45^hi^ CD11b^+^) in the brains of TMEV-infected mice at 3 and 7 d.p.i. **e** Percentage of TREM-1-expressing cells in the brains of TMEV-infected mice at 3 and 7 d.p.i. **f** Quantification of TREM-1-expressing macrophages in the brains of TMEV-infected mice at 3 and 7 d.p.i. **d**–**f** *n* = 6 mice per group; Student’s *t* test, *p < 0.05,**p < 0.01, df = 5. Gates were set according to FMO as described in the “Methods” section

Since *Trem*-*1* expression in the brains of TMEV-infected mice occurs specifically in infiltrating macrophages (Fig. 5b), we sought to confirm these results looking at TREM-1 protein expression by flow cytometry. C57BL/6 J mice were i.c. infected with TMEV and brains were harvested at 3 and 7 d.p.i. Cells were isolated and microglia, infiltrating macrophages, and lymphocytes were identified based on CD45 and CD11b levels of expression (Fig. 6a). TREM-1 staining suggested that TREM-1-positive cells were infiltrating macrophages (Fig. 6b). In order to confirm that infiltrating macrophages were the TREM-1 expressing cells in the CNS, we performed, on our flow cytometry plots, T-stochastic neighbor embedding (t-SNE) analysis (FlowJo) which highlights the relationship between cell clusters. We then overlaid each gated cell population manually, allowing us to clearly visualize microglia, infiltrating macrophages, and lymphocytes, demonstrating that indeed a specific subpopulation of infiltrating macrophages expressed TREM-1 (Fig. 6c, green population over blue). While there is a significantly higher percentage of infiltrating macrophages in the brains of TMEV-infected mice at 7 d.p.i. than at 3 d.p.i. (*p* < 0.01, df = 5, Student’s *t* test) (Fig. 6d), we found that the percentage of TREM-1-expressing macrophages was significantly increased at 3 d.p.i. compared to 7 d.p.i. (*p* < 0.05, df = 5, Student’s *t* test) (Fig. 6e) and the ratio of TREM-1/infiltrating macrophages was also significantly higher at 3 d.p.i. than at 7 d.p.i. (*p* < 0.01, df = 5, Student’s *t* test) (Fig. 6f). Taken together, our flow cytometry results show that TREM-1 expression is specific to infiltrating macrophages during virally induced neuroinflammation, thereby validating our RNA-Seq and qPCR data. Notably, since TREM-1 expression is correlated with induction of M1 macrophages and amplification of inflammatory cytokines [57, 58], lower expression of TREM-1 at day 7 compared to day 3 suggests a shift toward an anti-inflammatory phenotype. However, whether this alteration in the inflammatory response is associated with macrophage polarization or an increase in anti-inflammatory macrophages infiltrating into the CNS is currently unknown.

### Microglia and infiltrating macrophages display functional differences and similarities in the immune response gene profile

**Fig. 7:**
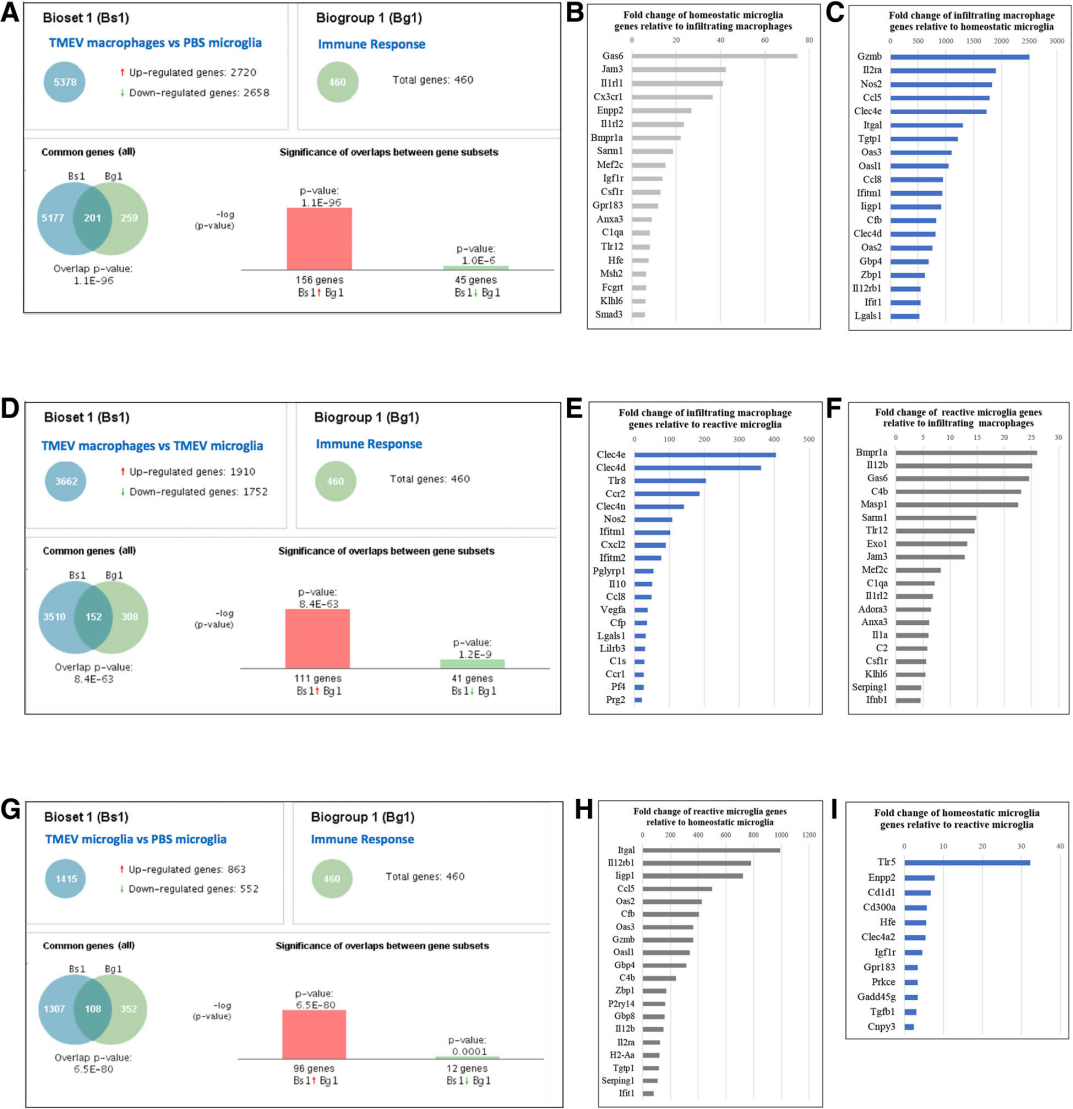
Immune response genes that are upregulated in microglia and infiltrating macrophages during neuroinflammation. Using the BaseSpace correlation engine, we identified **a** 201 immune response genes that were differentially expressed in the analysis comparing infiltrating macrophages from TMEV-infected brains vs homeostatic microglia from PBS-injected brains. Fold change of the 20 most highly enriched immune response genes in **b** homeostatic microglia relative to infiltrating macrophages; **c** infiltrating macrophages relative to homeostatic microglia. **d** 152 immune response genes that were differentially expressed in the analysis comparing infiltrating macrophages from TMEV-infected brains vs reactive microglia from TMEV-infected brains. Fold change of the 20 most highly enriched immune response genes in **e** infiltrating macrophages relative to reactive microglia; **f** reactive microglia relative to infiltrating macrophages. **g** 108 immune response genes that were differentially expressed in the analysis comparing reactive microglia from TMEV-infected brains vs homeostatic microglia from PBS-injected brains. **h** Fold change of the 20 most highly enriched immune response genes in reactive microglia relative to homeostatic microglia. **i** Fold change of the few enriched immune response genes found in homeostatic microglia relative to reactive microglia. All analyses were performed using the BaseSpace correlation engine

Microglia and infiltrating macrophages are both derived from the myeloid lineage and they perform a variety of functions in order to respond to CNS pathological insults and to maintain CNS homeostasis [8]. Proper activation and function of these cells are required in order to elicit innate and adaptive immune responses. Although both macrophages and microglia are part of the innate immunity, little is known about the similarities and differences between these two cell populations in the context of how they orchestrate immune responses. To gain insight into this question, we used the BaseSpace correlation engine to determine immune response genes that were upregulated in microglia and infiltrating macrophages during neuroinflammation. We first compared the gene expression profiles of infiltrating macrophages and homeostatic microglia to the immune response database, and we found 201 genes that overlapped with the immune response genes in the database (Fig. 7a). While expression of growth arrest specific 6 (*Gas6*), C-X3-C motif chemokine receptor 1 (*Cx3cr1*), and interleukin 1 receptor like 2 (*Il1rl2*) was enriched in homeostatic microglia relative to infiltrating macrophages (Fig. 7b), expression of granzyme B (*Gzmb*), interleukin 2 receptor subunit alpha (*Il2ra*), nitric oxide synthase 2 (*Nos2*), and 2′-5′-oligoadenylate synthetase 3 (*Oas3*) was specifically enriched in infiltrating macrophages relative to homeostatic microglia (Fig. 7c). Similarly, we compared the gene expression profiles of infiltrating macrophages and reactive microglia to the immune response database and found 152 genes that overlapped with the immune response genes in the database (Fig. 7d). *Clec4e*, C-type lectin domain family 4 member D (*Clec4d*), toll-like receptor 8 (*Tlr8*), and *Ccr2* were among the highly expressed genes enriched in infiltrating macrophages relative to reactive microglia (Fig. 7e). In contrast, we found the expression of bone morphogenetic protein receptor type 1A (*Bmpr1a*), interleukin 12B (*Il12b*), *Gas6*, and others to be enriched in reactive microglia relative to infiltrating macrophages (Fig. 7f). Lastly, we performed a similar analysis comparing the gene expression profiles of reactive microglia and homeostatic microglia to the immune response database (Fig. 7g). We found a total of 108 genes that overlapped with the immune response genes present in the database. Integrin subunit alpha L (*Itgal*), interleukin 12 receptor subunit beta 1 (*Il12rb1*), and C-C motif chemokine ligand 5 (*Ccl5*) were among the most highly differentially expressed genes in reactive microglia (Fig. 7h), whereas only a few genes were differentially expressed by homeostatic microglia (Fig. 7i). Since microglial activation is a hallmark of most neuroinflammatory diseases, genes that are highly expressed by these reactive cells might be useful biomarkers of neuroinflammation, particularly in the case of secreted proteins.

**Fig. 8:**
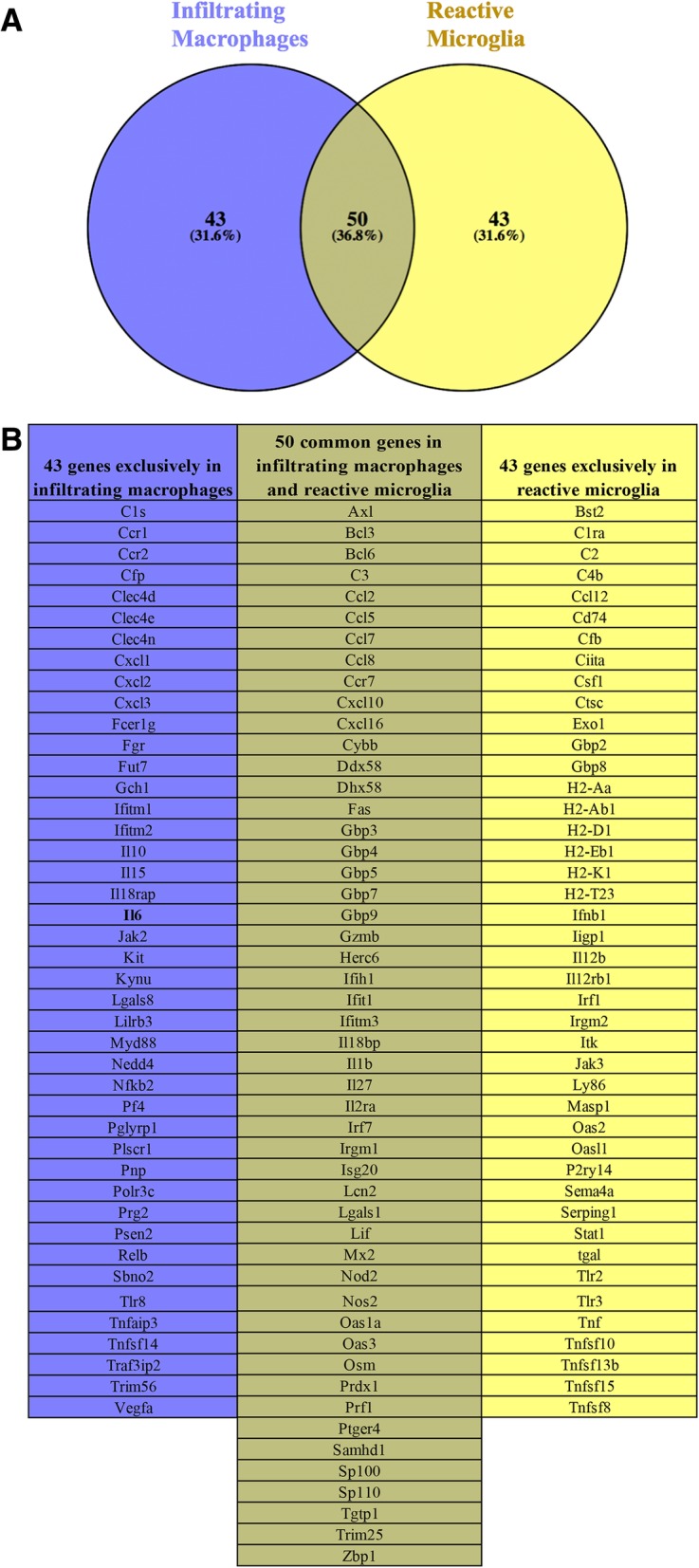
Functional differences and similarities in the microglial and macrophage immune response gene profiles during neuroinflammation. **a** Venn diagram of the immune response genes in infiltrating macrophages and reactive microglia that were identified in Fig. 7. Overlapping circles represent genes that are upregulated in both infiltrating macrophages and reactive microglia. **b** List of genes from the Venn diagram that are exclusively expressed by infiltrating macrophages (blue column), microglia (light yellow column), and by both cell populations (dark yellow column). Venn diagram was created using http://bioinfogp.cnb.csic.es/tools/venny/

In order to determine whether microglia and infiltrating macrophages have unique or redundant functions in immunity, we compared the immune-related gene expression profiles of these two cell populations. First, to determine immune genes that were uniquely upregulated by infiltrating macrophages, we compared the immune-related gene expression profile of infiltrating macrophages with that of homeostatic microglia and reactive microglia individually. Genes that were communal in both comparisons were selected as infiltrating macrophage (total of 93). Second, to determine genes that were expressed by reactive microglia, we compared genes that were upregulated by reactive microglia compared to homeostatic microglia (total of 93). Using a Venn diagram, we found 43 immune genes that were specifically expressed by infiltrating macrophages, 43 immune genes that were uniquely expressed by reactive microglia, and 50 immune genes that were commonly expressed in both infiltrating macrophages and reactive microglia (Fig. 8a). A comprehensive list of these genes is given in Fig. 8b. Although MHC-II genes are shown to be uniquely expressed by reactive microglia, this is a result of the biases of our analysis method. MHC-II genes are induced in infiltrating macrophages when compared to homeostatic microglia (Fig. 7h). This result suggests that microglia and infiltrating macrophages share common functions in the context of the immune response but also that these cells display specialization, as demonstrated by genes that are specifically expressed in one cell population or the other.

## Discussion

This study differs in several ways from valuable recently published works on microglia and infiltrating macrophages [27, 31, 59–61]. First, we isolated cells from the brains of mice during neuroinflammation induced by a viral infection. Second, microglia and infiltrating macrophages were both isolated from the CNS of virally infected mice instead of using macrophages obtained from the periphery or macrophages in vitro-derived from bone marrow. Since the tissue environment plays an important role in shaping both the phenotype and function of macrophages, it stands to reason that it also modulates the gene expression profile of macrophages. Therefore, we would argue that it is particularly important, when comparing these two cell populations, to obtain them from the same tissue and, if possible, the same mouse. Third, besides just describing our RNA-Seq results and Log2 fold changes, we have also confirmed and quantified the expression of our candidate genes by qPCR. Fourth, we confirmed the specificity of the expression of particular genes in infiltrating macrophages or microglia (in homeostatic and reactive states) by also analyzing gene expression in lymphocytes isolated from brains during neuroinflammation, and/or in splenic T cells isolated from TMEV-infected and PBS-injected mice. Not surprisingly, some of our candidate genes, when analyzed by qPCR, were found to also be expressed in cells other than infiltrating macrophages and microglia (Additional file 1: Figure S1B).

Although an exceptional study performed under physiological and aging conditions highlighted genes used by microglia for sensing changes in the CNS [62], a comparison between microglial and macrophage “sensomes” during neuroinflammation had not yet been performed. Here, we have found that reactive microglia and infiltrating macrophages have both redundant and also distinct functions in relation to the expression of immune genes during virally induced neuroinflammation. One of the most important mediators of inflammation is cytokines. We found that both cell populations shared in their ability to express interleukins *Il*-*1b* and *Il*-*27*. While IL-1b played a role in promoting inflammatory reaction in the CNS, IL-27 seemed to antagonize the inflammatory response by promoting IL-10 production (reviewed in [63]). Importantly, administration of IL-27 inhibited neuroinflammation and modulated disease development in an experimental autoimmune encephalomyelitis (EAE) model of demyelinating disease [64]. Chemokine ligands, such as *Ccl2*, *Ccl5*, *Ccl7*, and *Ccl8*, as well as *Cxcl10* and *Cxcl16*, were also commonly expressed by infiltrating macrophages and microglia. CCL2 and CCL7 proteins were required for efficient infiltration of macrophages into the CNS [65], while CXCL10 played a role in the recruitment of lymphocytes from the periphery, mediating CNS inflammation. Furthermore, CCL5 modulated microglial activation toward an inflammatory state, increasing the production of nitric oxide and inhibiting the anti-inflammatory cytokine IL-10 [66]. We also found that the genes for guanylate-binding proteins (*Gbp3*, *Gbp4*, *Gbp5*, G*bp7*, and *Gbp9*) were commonly expressed by reactive microglia and infiltrating macrophages. Gbps are a family of GTPase proteins induced by interferon which critically orchestrate the innate immune response.

We found that complement genes such as *C1ra*, *C2*, *C4b*, and *Masp1* were expressed by reactive microglia. Activation of the complement system in the CNS can result in substantial secretion of inflammatory molecules resulting in neuroinflammation [67]. Furthermore, microglia were demonstrated to be a source for Sema4A in the CNS, and higher levels of Sema4A were found in multiple sclerosis plaques compared to control tissue [68], suggesting a role for this protein in neuroinflammation. Earlier published studies demonstrated that TNF was specifically expressed by reactive microglia but not by infiltrating macrophages in the CNS [15, 69]. Our analyses confirmed that *Tnf* was indeed found in microglia but not in macrophages, highlighting the specific induction of immune genes by these two different myeloid cells. Importantly, while low levels of TNF were important to maintain homeostatic functions within the CNS, increased expression of TNF was often associated with inflammation [70], as observed in many neuroinflammatory conditions such as Alzheimer’s disease, seizures, traumatic brain injury, and others [ [71], reviewed in [72]].

Interestingly, our analysis also demonstrated that *Il*-*6* was specifically expressed by infiltrating macrophages, but not by reactive microglia, in the brains of infected mice. This agrees with previously published work from our lab, where we demonstrated, through chimeric mice, that IL-6 cytokine in the brain of TMEV-infected mice was mostly produced by infiltrating macrophages [15]. Like TNF, increased levels of IL-6 in the CNS were seen during neuroinflammatory conditions [73]. Besides *Il*-*6*, *Il*-*10* and *Il*-*15* were found to be exclusively expressed by infiltrating macrophages. We also found that C-type lectins, such as *Clec4d*, *Clec4e*, and *Clec4n*, were expressed by infiltrating macrophages. Importantly, *Mincle* (*Clec4e*) has been suggested to play a role in the recruitment of inflammatory cells into the CNS [74, 75].

Our search for microglial- and macrophage-specific cell surface markers through RNA-Seq revealed that *P2yr12*, *Mrc2*, and *Pros1* were specifically expressed by microglia (Fig. 5a). These are known to be highly expressed microglial genes and the presence of these genes specifically in microglia validates our assay. In addition, our results identified previously unreported cell surface molecules that were uniquely expressed by microglia, such as *Sdk1*, *Ecscr*, *Sgce*, *Slc12a2*, *Sema4g*, *Smo*, *Ptprm*, *Kcnd1*, *Slco2b1*, *Slc46a1*, *Jam2*, and *Plxna4* (Fig. 5a and Additional file 1: Figure S1A). Interestingly, homeostatic microglia expressed higher levels of most of the genes analyzed in this study compared to reactive microglia, and further studies will be required to determine the relevant function of these proteins in the context of microglial biology. Additionally, we showed for the first time, that in addition to *Msr1* (previously shown to be highly expressed in infiltrating macrophages compared to microglia [55]), *Slfn4*, *Ms4a8a*, *Trem-1*, *Ifitm6*, *Lilra6*, *Sirpb1a*, and *Clec4e* were all specifically expressed by infiltrating macrophages in the brain during neuroinflammation (Fig. 5b). These findings demonstrate new cell surface molecules that can potentially be used as markers to discriminate between microglia and infiltrating macrophages.

With a total of 18 members [76], the MS4A family of proteins is characterized by a predicted four transmembrane helix-spanning structure, with amino- and carboxy-terminal cytoplasmic domains. Although MS4A proteins have been suggested to function as cell surface signaling molecules and intracellular adaptor proteins, their function is not fully understood [76–78]. Importantly, some members of the MS4A family have been associated with several diseases such as atopy, Alzheimer’s disease, and arthritis [79, 80]. Recently, membrane-spanning 4-domains, subfamily A, member 7 (*Ms4a7*) was shown to be expressed in microglia and brain border macrophages [55, 59, 60]. Similarly, in our study, we found *Ms4a7* (data not shown) and *Ms4a6d* (Additional file 1: Figure S1B) expression in the microglia and infiltrating macrophages from TMEV-infected mouse brains. We also showed, for the first time, that another member of the MS4A family, *Ms4a8a*, was predominantly expressed in macrophages that infiltrate the CNS but not in microglia (Fig. 5b). Interestingly, MS4A8A was identified as a protein expressed by tumor-associated macrophages and its expression seemed to be a marker for M2 macrophages [54, 78]. Although further studies will be needed to understand the exact function of these proteins, the results presented here suggest that specific MS4A proteins might play distinct roles in infiltrating macrophages and microglia during neuroinflammation.

Our flow cytometry analysis confirmed that TREM-1 was uniquely expressed by infiltrating macrophages in the CNS (Fig. 6). TREM-1 is a cell surface receptor from the TREM family, which is composed of activating and inhibitory receptors [81, 82]. TREM-2, which was initially characterized as an inhibitory receptor, is expressed by microglia [62, 83–85], whereas TREM-1 is involved in activation and amplification of the inflammatory immune response and is expressed in myeloid cells [86, 87]. TREM-1 can be found in two different forms: membrane-bound and as a soluble receptor (sTREM-1). A growing body of literature supports a role for TREM-1 in several inflammatory diseases, such as atherosclerosis, myocarditis, diabetes, and others [58, 80, 88, 89]. TREM-1 activation was correlated with induction and amplification of proinflammatory cytokines, such as IL-6 and TNF-α [90]. In our model of virus-induced seizures/epilepsy, we found that IL-6 played a major role in acute seizures, and infiltrating macrophages were the major producers of IL-6. Furthermore, we found that TREM-1 was expressed by a subset of the infiltrating macrophages and TREM-1 expression was higher at 3 d.p.i. than at 7 d.p.i. in the brains of TMEV-infected mice (Fig. 6). Although we are showing, for the first time, that TREM-1 is found in macrophages that infiltrate the brain, its expression in peripheral macrophages has already been demonstrated [57, 58, 87]. Furthermore, since induction and amplification of the inflammatory immune response were functions attributed to TREM-1, we hypothesized that TREM-1 expression correlated with the presence of M1 (inflammatory) macrophages. This idea supports the fact that in TMEV-infected mice, we expect to see an increased level of M1 macrophages at day 3, which is the beginning of acute seizures. Although additional studies are required to better understand the function of this protein in brain-infiltrating macrophages, we propose that TREM-1 expression in macrophages in the CNS could be used as a maker of inflammatory (M1) macrophages and its presence in the brain may also be a marker for neuroinflammation.

## Conclusions

In summary, our work validated previously unknown markers that could be used to discriminate microglia from infiltrating macrophages during viral-induced neuroinflammation. Despite the fact that further studies are required to extensively explore the results presented here, our analysis comparing immune-induced genes in microglia and infiltrating macrophages is especially important since it can potentially be utilized to identify biomarkers of neuroinflammation. Furthermore, modulation of macrophage recruitment to the CNS, as well as modulation of microglia and macrophage inflammatory states, may be a promising therapeutic strategy to control neuroinflammation and to treat neuroinflammatory disorders. Our findings can be further exploited in order to specifically immunomodulate cell responses that are induced upon neuroinflammation.

## Additional files

Additional file 1: **Figure S1.** Validation of gene expression of microglial- and macrophage-specific genes. (DOCX 354 kb)

Additional file 2: **Table S1.** List of primers used for qPCR. (DOCX 17 kb)

## Abbreviations

APC: Allophycocyanin
Arg2: Arginase, type II
Bmpr1a: Bone morphogenetic protein receptor type 1A
BV: Brilliant Violet
Ccl5: C-C motif chemokine ligand 5
Ccr2: C-C motif chemokine receptor 2
CCS: Cosmic calf serum
Clec4d: C-type lectin domain family 4, member D
Clec4e: C-type lectin domain family 4, member E
CNS: Central nervous system
Cx3cr1: C-X3-C motif chemokine receptor 1
d.p.i.: Days post infection
DA: Daniels
Ecscr: Endothelial cell surface expressed chemotaxis and apoptosis regulator
Fcrls: Fc receptor-like S
FMO: Fluorescence-minus-one
Gas6: Growth arrest specific 6
Gbp: Guanylate-binding proteins
GPCRs: G protein-coupled receptors
GSEA: Gene set enrichment analysis
Gzmb: Granzyme B
i.c.: Intracerebral
Iba1: Ionized calcium-binding adapter molecule 1
Ifi204: Interferon-activated gene 204
Ifitm6: Interferon-induced transmembrane protein 6
IL: Interleukin
Il12b: Interleukin 12B
Il12rb1: Interleukin 12 receptor subunit beta 1
Il1rl2: Interleukin 1 receptor like 2
Il2ra: Interleukin 2 receptor subunit alpha
Itgal: Integrin subunit alpha L
Jam2: Junctional adhesion molecule 2
JHMV: JHM strain of mouse hepatitis virus
Kcnd1: Potassium voltage-gated channel subfamily D member 1
Kcnn4: Potassium calcium-activated channel subfamily N member 4
Lilra6: Leukocyte immunoglobulin-like receptor subfamily A, member 6
Ly6c2: Lymphocyte antigen 6 complex, locus C2
Lyz2: Lysozyme 2
MHC-II: Major histocompatibility complex class-II
Mrc2: Mannose receptor C type 2
Ms4a6d: Membrane-spanning 4-domains, subfamily A, member 6A
Ms4a7: Membrane-spanning 4-domains, subfamily A, member 7
Ms4a8a: Membrane-spanning 4-domains, subfamily A, member 8A
Msr1: Macrophage scavenger receptor 1
Msrb1: Methionine sulfoxide reductase
Nos2: Nitric oxide synthase 2
Oas3: 2′-5′-Oligoadenylate synthetase 3
P2ry12: P2Y purinoceptor 12
PBS: Phosphate-buffered saline
PCA: Principal component analysis
PE: Phycoerythrin
PFU: Plaque-forming units
Plxna4: Plexin A4
Pros1: Protein S
Ptprm: Protein tyrosine phosphatase, receptor type M
qPCR: Quantitative PCR
RNA-Seq: RNA sequencing
S100a10: S100 calcium binding protein A10
Sall1: Spalt-like transcription factor 1
Sdk1: Sidekick cell adhesion molecule 1
Sema4g: Semaphorin 4G
Sgce: Sarcoglycan epsilon
SiglecH: Sialic acid-binding immunoglobulin-type lectin H
Sirpb1a: SIRP-beta B-type
Slc12a2: Solute carrier family 12, member 2
Slc2a5: Solute carrier family 2, facilitated glucose transporter member 5
Slc46a1: Solute carrier family 46, member 1
Slco2b1: Solute carrier organic anion transporter family, member 2B1
Slfn4: Schlafen 4
Smo: Smoothened homolog
sTREM-1: Soluble TREM-1
Tlr8: Toll-like receptor 8
TMEM119: Transmembrane protein 119
TMEV: Theiler’s murine encephalomyelitis virus
TNF: Tumor necrosis factor
Trem-1: Triggering receptor expressed on myeloid cells 1
t-SNE: T-stochastic neighbor embedding
Vim: Vimentin

## References

[1] Gomez Perdiguero E, Schulz C, Geissmann F (2013). Development and homeostasis of “resident” myeloid cells: the case of the microglia. Glia..

[2] Lawson LJ, Perry VH, Dri P, Gordon S (1990). Heterogeneity in the distribution and morphology of microglia in the normal adult mouse brain. Neuroscience..

[3] Ginhoux F, Greter M, Leboeuf M, Nandi S, See P, Gokhan S (2010). Fate mapping analysis reveals that adult microglia derive from primitive macrophages. Science..

[4] Hoeffel G, Chen J, Lavin Y, Low D, Almeida FF, See P (2015). C-Myb(+) erythro-myeloid progenitor-derived fetal monocytes give rise to adult tissue-resident macrophages. Immunity..

[5] Cronk JC, Kipnis J (2013). Microglia—the brain's busy bees. F1000Prime Rep.

[6] Sousa C, Biber K, Michelucci A (2017). Cellular and molecular characterization of microglia: a unique immune cell population. Front Immunol.

[7] Subramaniam SR, Federoff HJ (2017). Targeting microglial activation states as a therapeutic avenue in Parkinson's disease. Front Aging Neurosci.

[8] Yin J, Valin KL, Dixon ML, Leavenworth JW (2017). The role of microglia and macrophages in CNS homeostasis, autoimmunity, and cancer. J Immunol Res.

[9] Mathys H, Adaikkan C, Gao F, Young JZ, Manet E, Hemberg M (2017). Temporal tracking of microglia activation in neurodegeneration at single-cell resolution. Cell Rep.

[10] Boche D, Perry VH, Nicoll JA (2013). Review: activation patterns of microglia and their identification in the human brain. Neuropathol Appl Neurobiol.

[11] Hayes GM, Woodroofe MN, Cuzner ML (1987). Microglia are the major cell type expressing MHC class II in human white matter. J Neurol Sci.

[12] Hayes GM, Woodroofe MN, Cuzner ML (1988). Characterisation of microglia isolated from adult human and rat brain. J Neuroimmunol.

[13] Greter M (2016). Family ties among CNS macrophages. Nat Immunol.

[14] London A, Cohen M, Schwartz M (2013). Microglia and monocyte-derived macrophages: functionally distinct populations that act in concert in CNS plasticity and repair. Front Cell Neurosci.

[15] Cusick MF, Libbey JE, Patel DC, Doty DJ, Fujinami RS (2013). Infiltrating macrophages are key to the development of seizures following virus infection. J Virol.

[16] Miron VE, Franklin RJ (2014). Macrophages and CNS remyelination. J Neurochem.

[17] Katsumoto A, Lu H, Miranda AS, Ransohoff RM (2014). Ontogeny and functions of central nervous system macrophages. J Immunol.

[18] Li Q, Barres BA (2018). Microglia and macrophages in brain homeostasis and disease. Nat Rev Immunol.

[19] Parisi L, Gini E, Baci D, Tremolati M, Fanuli M, Bassani B (2018). Macrophage polarization in chronic inflammatory diseases: killers or builders?. J Immunol Res.

[20] Blaylock RL (2017). Parkinson's disease: microglial/macrophage-induced immunoexcitotoxicity as a central mechanism of neurodegeneration. Surg Neurol Int.

[21] Chu F, Shi M, Zheng C, Shen D, Zhu J, Zheng X (2018). The roles of macrophages and microglia in multiple sclerosis and experimental autoimmune encephalomyelitis. J Neuroimmunol.

[22] DePaula-Silva AB, Sonderegger FL, Libbey JE, Doty DJ, Fujinami RS (2018). The immune response to picornavirus infection and the effect of immune manipulation on acute seizures. J Neurovirol.

[23] Kumar A, Alvarez-Croda DM, Stoica BA, Faden AI, Loane DJ (2016). Microglial/macrophage polarization dynamics following traumatic brain injury. J Neurotrauma.

[24] Moehle MS, West AB (2015). M1 and M2 immune activation in Parkinson's disease: foe and ally?. Neuroscience..

[25] Wu SY, Watabe K (2017). The roles of microglia/macrophages in tumor progression of brain cancer and metastatic disease. Front Biosci (Landmark Ed).

[26] Greter M, Lelios I, Croxford AL (2015). Microglia versus myeloid cell nomenclature during brain inflammation. Front Immunol.

[27] Bennett ML, Bennett FC, Liddelow SA, Ajami B, Zamanian JL, Fernhoff NB (2016). New tools for studying microglia in the mouse and human CNS. Proc Natl Acad Sci U S A.

[28] Butovsky O, Jedrychowski MP, Moore CS, Cialic R, Lanser AJ, Gabriely G (2014). Identification of a unique TGF-beta-dependent molecular and functional signature in microglia. Nat Neurosci.

[29] Zrzavy T, Hametner S, Wimmer I, Butovsky O, Weiner HL, Lassmann H (2017). Loss of ‘homeostatic’ microglia and patterns of their activation in active multiple sclerosis. Brain.

[30] Mildner A, Huang H, Radke J, Stenzel W, Priller J (2017). P2Y12 receptor is expressed on human microglia under physiological conditions throughout development and is sensitive to neuroinflammatory diseases. Glia..

[31] Savarin C, Dutta R, Bergmann CC (2018). Distinct gene profiles of bone marrow-derived macrophages and microglia during neurotropic coronavirus-induced demyelination. Front Immunol.

[32] Lund H, Pieber M, Harris RA (2017). Lessons learned about neurodegeneration from microglia and monocyte depletion studies. Front Aging Neurosci.

[33] Waisman A, Ginhoux F, Greter M, Bruttger J (2015). Homeostasis of microglia in the adult brain: review of novel microglia depletion systems. Trends Immunol.

[34] DePaula-Silva AB, Hanak TJ, Libbey JE, Fujinami RS (2017). Theiler's murine encephalomyelitis virus infection of SJL/J and C57BL/6J mice: models for multiple sclerosis and epilepsy. J Neuroimmunol.

[35] Libbey JE, Fujinami RS (2011). Neurotropic viral infections leading to epilepsy: focus on Theiler's murine encephalomyelitis virus. Futur Virol.

[36] Libbey JE, Kirkman NJ, Smith MC, Tanaka T, Wilcox KS, White HS (2008). Seizures following picornavirus infection. Epilepsia..

[37] Waltl I, Kaufer C, Gerhauser I, Chhatbar C, Ghita L, Kalinke U (2018). Microglia have a protective role in viral encephalitis-induced seizure development and hippocampal damage. Brain Behav Immun.

[38] Wheeler DL, Sariol A, Meyerholz DK, Perlman S (2018). Microglia are required for protection against lethal coronavirus encephalitis in mice. J Clin Invest.

[39] Sanchez JMS, DePaula-Silva AB, Doty DJ, Truong A, Libbey JE, Fujinami RS. Microglial cell depletion is fatal with low level picornavirus infection of the central nervous system. J Neurovirol (in Press). 2019; 10.1007/s13365-019-00740-3 PMC6635090 30859497

[40] Tsunoda I, McCright IJ, Kuang LQ, Zurbriggen A, Fujinami RS (1997). Hydrocephalus in mice infected with a Theiler's murine encephalomyelitis virus variant. J Neuropathol Exp Neurol.

[41] Gorbea C, Mosbruger T, Cazalla D (2017). A viral Sm-class RNA base-pairs with mRNAs and recruits microRNAs to inhibit apoptosis. Nature..

[42] Dobin A, Davis CA, Schlesinger F, Drenkow J, Zaleski C, Jha S (2013). STAR: ultrafast universal RNA-seq aligner. Bioinformatics..

[43] Martin M (2011). Cutadapt removes adapter sequences from. EMBnet J.

[44] Liao Y, Smyth GK, Shi W (2014). featureCounts: an efficient general purpose program for assigning sequence reads to genomic features. Bioinformatics..

[45] Ewels P, Magnusson M, Lundin S, Kaller M (2016). MultiQC: summarize analysis results for multiple tools and samples in a single report. Bioinformatics..

[46] Love MI, Huber W, Anders S (2014). Moderated estimation of fold change and dispersion for RNA-seq data with DESeq2. Genome Biol.

[47] Mootha VK, Lindgren CM, Eriksson KF, Subramanian A, Sihag S, Lehar J (2003). PGC-1alpha-responsive genes involved in oxidative phosphorylation are coordinately downregulated in human diabetes. Nat Genet.

[48] Subramanian A, Tamayo P, Mootha VK, Mukherjee S, Ebert BL, Gillette MA (2005). Gene set enrichment analysis: a knowledge-based approach for interpreting genome-wide expression profiles. Proc Natl Acad Sci U S A.

[49] Ford AL, Goodsall AL, Hickey WF, Sedgwick JD (1995). Normal adult ramified microglia separated from other central nervous system macrophages by flow cytometric sorting. Phenotypic differences defined and direct ex vivo antigen presentation to myelin basic protein-reactive CD4+ T cells compared. J Immunol.

[50] Cucak H, Grunnet LG, Rosendahl A (2014). Accumulation of M1-like macrophages in type 2 diabetic islets is followed by a systemic shift in macrophage polarization. J Leukoc Biol.

[51] Phipps KD, Surette AP, O'Connell PA, Waisman DM (2011). Plasminogen receptor S100A10 is essential for the migration of tumor-promoting macrophages into tumor sites. Cancer Res.

[52] Lee BC, Lee SG, Choo MK, Kim JH, Lee HM, Kim S (2017). Selenoprotein MsrB1 promotes anti-inflammatory cytokine gene expression in macrophages and controls immune response in vivo. Sci Rep.

[53] Sigalov AB (2014). A novel ligand-independent peptide inhibitor of TREM-1 suppresses tumor growth in human lung cancer xenografts and prolongs survival of mice with lipopolysaccharide-induced septic shock. Int Immunopharmacol.

[54] Weitnauer M, Schmidt L, Ng Kuet Leong N, Muenchau S, Lasitschka F, Eckstein V (2014). Bronchial epithelial cells induce alternatively activated dendritic cells dependent on glucocorticoid receptor signaling. J Immunol.

[55] Buttgereit A, Lelios I, Yu X, Vrohlings M, Krakoski NR, Gautier EL (2016). Sall1 is a transcriptional regulator defining microglia identity and function. Nat Immunol.

[56] Konishi H, Kobayashi M, Kunisawa T, Imai K, Sayo A, Malissen B (2017). Siglec-H is a microglia-specific marker that discriminates microglia from CNS-associated macrophages and CNS-infiltrating monocytes. Glia..

[57] Lo TH, Tseng KY, Tsao WS, Yang CY, Hsieh SL, Chiu AW (2014). TREM-1 regulates macrophage polarization in ureteral obstruction. Kidney Int.

[58] Subramanian S, Pallati PK, Sharma P, Agrawal DK, Nandipati KC (2017). TREM-1 associated macrophage polarization plays a significant role in inducing insulin resistance in obese population. J Transl Med.

[59] Hammond TR, Dufort C, Dissing-Olesen L, Giera S, Young A, Wysoker A (2019). Single-cell RNA sequencing of microglia throughout the mouse lifespan and in the injured brain reveals complex cell-state changes. Immunity..

[60] MJC J, Sankowski R, Brendecke SM, Sagar LG, Tai YH, et al. Single-cell profiling identifies myeloid cell subsets with distinct fates during neuroinflammation. Science. 2019;363(6425) Epub 2019/01/27 10.1126/science.aat7554 30679343

[61] Lewis ND, Hill JD, Juchem KW, Stefanopoulos DE, Modis LK (2014). RNA sequencing of microglia and monocyte-derived macrophages from mice with experimental autoimmune encephalomyelitis illustrates a changing phenotype with disease course. J Neuroimmunol.

[62] Hickman SE, Kingery ND, Ohsumi TK, Borowsky ML, Wang LC, Means TK (2013). The microglial sensome revealed by direct RNA sequencing. Nat Neurosci.

[63] Yoshida H, Hunter CA (2015). The immunobiology of interleukin-27. Annu Rev Immunol.

[64] Casella G, Finardi A, Descamps H, Colombo F, Maiorino C, Ruffini F (2017). IL-27, but not IL-35, inhibits neuroinflammation through modulating GM-CSF expression. Sci Rep.

[65] Bardina SV, Michlmayr D, Hoffman KW, Obara CJ, Sum J, Charo IF (2015). Differential roles of chemokines CCL2 and CCL7 in Monocytosis and leukocyte migration during West Nile virus infection. J Immunol.

[66] Skulina C, Schmidt S, Dornmair K, Babbe H, Roers A, Rajewsky K (2004). Multiple sclerosis: brain-infiltrating CD8+ T cells persist as clonal expansions in the cerebrospinal fluid and blood. Proc Natl Acad Sci U S A.

[67] Orsini F, De Blasio D, Zangari R, Zanier ER, De Simoni MG (2014). Versatility of the complement system in neuroinflammation, neurodegeneration and brain homeostasis. Front Cell Neurosci.

[68] Leitner Dominique F., Todorich Bozho, Zhang Xuesheng, Connor James R. (2015). Semaphorin4A Is Cytotoxic to Oligodendrocytes and Is Elevated in Microglia and Multiple Sclerosis. ASN Neuro.

[69] Welser-Alves JV, Milner R (2013). Microglia are the major source of TNF-alpha and TGF-beta1 in postnatal glial cultures; regulation by cytokines, lipopolysaccharide, and vitronectin. Neurochem Int.

[70] Olmos G, Llado J (2014). Tumor necrosis factor alpha: a link between neuroinflammation and excitotoxicity. Mediat Inflamm.

[71] Patel Dipan C., Wallis Glenna, Dahle E. Jill, McElroy Pallavi B., Thomson Kyle E., Tesi Raymond J., Szymkowski David E., West Peter J., Smeal Roy M., Patel Manisha, Fujinami Robert S., White H. Steve, Wilcox Karen S. (2017). Hippocampal TNFα Signaling Contributes to Seizure Generation in an Infection-Induced Mouse Model of Limbic Epilepsy. eneuro.

[72] Clark IA, Vissel B (2016). Excess cerebral TNF causing glutamate excitotoxicity rationalizes treatment of neurodegenerative diseases and neurogenic pain by anti-TNF agents. J Neuroinflammation.

[73] Erta M, Quintana A, Hidalgo J (2012). Interleukin-6, a major cytokine in the central nervous system. Int J Biol Sci.

[74] Arumugam TV, Manzanero S, Furtado M, Biggins PJ, Hsieh YH, Gelderblom M (2017). An atypical role for the myeloid receptor mincle in central nervous system injury. J Cereb Blood Flow Metab.

[75] Patin EC, Orr SJ, Schaible UE (2017). Macrophage inducible C-type lectin as a multifunctional player in immunity. Front Immunol.

[76] Eon Kuek L, Leffler M, Mackay GA, Hulett MD (2016). The MS4A family: counting past 1, 2 and 3. Immunol Cell Biol.

[77] Liang Y, Tedder TF (2001). Identification of a CD20-, FcepsilonRIbeta-, and HTm4-related gene family: sixteen new MS4A family members expressed in human and mouse. Genomics..

[78] Michel J, Schonhaar K, Schledzewski K, Gkaniatsou C, Sticht C, Kellert B (2013). Identification of the novel differentiation marker MS4A8B and its murine homolog MS4A8A in colonic epithelial cells lost during neoplastic transformation in human colon. Cell Death Dis.

[79] Fujikado N, Saijo S, Iwakura Y (2006). Identification of arthritis-related gene clusters by microarray analysis of two independent mouse models for rheumatoid arthritis. Arthritis Res Ther.

[80] Proitsi P, Lee SH, Lunnon K, Keohane A, Powell J, Troakes C (2014). Alzheimer's disease susceptibility variants in the MS4A6A gene are associated with altered levels of MS4A6A expression in blood. Neurobiol Aging.

[81] Arts RJ, Joosten LA, van der Meer JW, Netea MG (2013). TREM-1: intracellular signaling pathways and interaction with pattern recognition receptors. J Leukoc Biol.

[82] Klesney-Tait J, Turnbull IR, Colonna M (2006). The TREM receptor family and signal integration. Nat Immunol.

[83] Kiialainen A, Hovanes K, Paloneva J, Kopra O, Peltonen L (2005). Dap12 and Trem2, molecules involved in innate immunity and neurodegeneration, are co-expressed in the CNS. Neurobiol Dis.

[84] Schmid CD, Sautkulis LN, Danielson PE, Cooper J, Hasel KW, Hilbush BS (2002). Heterogeneous expression of the triggering receptor expressed on myeloid cells-2 on adult murine microglia. J Neurochem.

[85] Sessa G, Podini P, Mariani M, Meroni A, Spreafico R, Sinigaglia F (2004). Distribution and signaling of TREM2/DAP12, the receptor system mutated in human polycystic lipomembraneous osteodysplasia with sclerosing leukoencephalopathy dementia. Eur J Neurosci.

[86] Bouchon A, Dietrich J, Colonna M (2000). Cutting edge: inflammatory responses can be triggered by TREM-1, a novel receptor expressed on neutrophils and monocytes. J Immunol.

[87] Bouchon A, Facchetti F, Weigand MA, Colonna M (2001). TREM-1 amplifies inflammation and is a crucial mediator of septic shock. Nature..

[88] Ford JW, McVicar DW (2009). TREM and TREM-like receptors in inflammation and disease. Curr Opin Immunol.

[89] Klesney-Tait J, Keck K, Li X, Gilfillan S, Otero K, Baruah S (2013). Transepithelial migration of neutrophils into the lung requires TREM-1. J Clin Invest.

[90] Netea MG, Azam T, Ferwerda G, Girardin SE, Kim SH, Dinarello CA (2006). Triggering receptor expressed on myeloid cells-1 (TREM-1) amplifies the signals induced by the NACHT-LRR (NLR) pattern recognition receptors. J Leukoc Biol.

