# Supplementary material for: Differential transcriptional profiles identify microglial- and macrophage-specific gene markers expressed during virus-induced neuroinflammation
Source: J Neuroinflammation. 2019 Jul 20;16:152. doi: 10.1186/s12974-019-1545-x (PMC6642742; doi:10.1186/s12974-019-1545-x)
Supplement: Supplementary file 1 — Figure S1. Validation of gene expression of microglial- and macrophage-specific genes. (DOCX 354 kb) [file 12974_2019_1545_MOESM1_ESM.docx]

**Additional file 1: Figure S1**


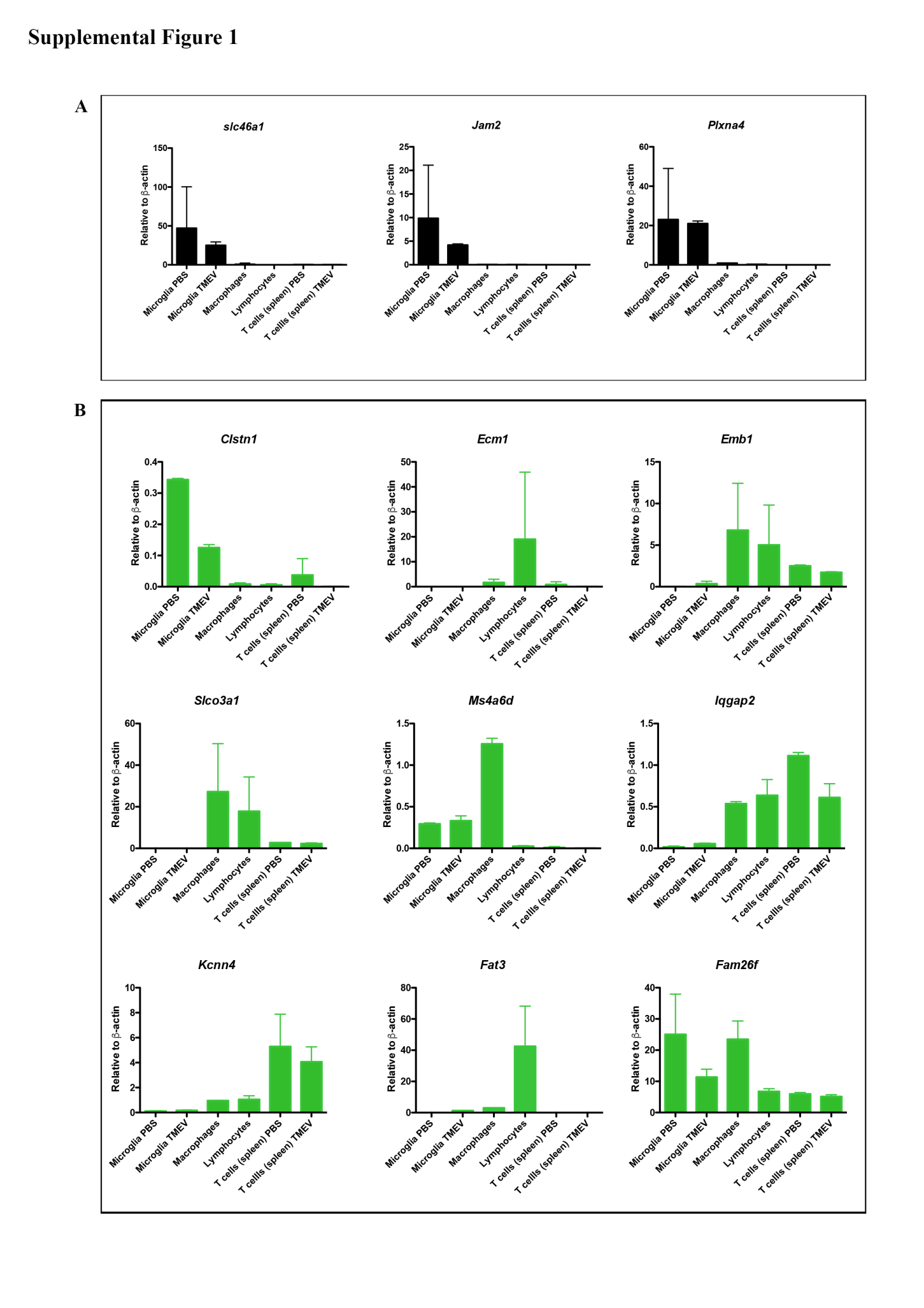


**Figure S1:** **Validation of gene expression of microglial- and macrophage-specific genes**. **(A)** Level of mRNA expression of microglial-specific genes. **(B)** Level of mRNA expression of genes that we found expressed in different cells other than macrophages and microglia. Cells obtained from the brains of TMEV-infected (n = 10) and PBS-injected (n = 20) mice, and from spleens of TMEV-infected (n = 5) and PBS-injected (n = 5) mice were cell sorted as follows: microglia (CD45^lo/int^ CD11b^+^), macrophages (CD45^hi^ CD11b^+^), brain lymphocytes (CD45^+^ CD11b^lo/int^), and splenic T cells (CD45^+^ CD3^+^). RNA was extracted and quantified by qPCR. Levels were normalized to β-actin. Data are presented as means ± standard deviation. Shown is one of two independent experiments.
