# Supplementary material for: Differential transcriptional profiles identify microglial- and macrophage-specific gene markers expressed during virus-induced neuroinflammation
Source: J Neuroinflammation. 2019 Jul 20;16:152. doi: 10.1186/s12974-019-1545-x (PMC6642742; doi:10.1186/s12974-019-1545-x)
Supplement: Supplementary file 2 — Table S1. List of primers used for qPCR. (DOCX 17 kb) [file 12974_2019_1545_MOESM2_ESM.docx]

**Additional file 2: Table S1** List of primers used for qPCR

| **Gene Symbol** | **Primer Sequences** |
| --- | --- |
| ***β-actin*** | FWD: AGGGAAATCGTGCGTGACAT REV: GAACCGCTCGTTGCCAATAG |
| ***Agmo*** | FWD: ACCAAAGATCCAAGGCAGCTA REV: CCAAGAGCCAGAGGTGAGTT |
| ***Alcam*** | FWD: GGGAGCGTCATAAACCAAACAG REV: TATCGTCTGCCTCATCGTGC |
| ***Antxr2*** | FWD: GAAGGTGATGAGGGCCGATG REV: GGGCTGTTGTCCCAAGAACT |
| ***Bst1*** | FWD: GCCTATCCCACGAGAGGGTTT  REV: ATTGGGCCCTCCAACGTCAT |
| ***Cadm1*** | FWD: AACATGCCGTACTGTCTGGG REV: CGCCGTGGTGTCGTATACAT |
| ***Clec4e*** | FWD: TTTAAAAAGAGGGCCAAGGATTC REV: TGAAGCATCCTCTCTCTGTGTG |
| ***Clstn1*** | FWD: ACAGTCACCAAAGAAGGTGAGA REV: TCCCTCACCCGTGGACTTAT |
| ***Cmtm4*** | FWD: CATCTGTATCCGAGCCCCAC REV: GCCGACAACACTTCCTCAGA |
| ***Ecm1*** | FWD: ACAAAGAGAAGTGCAGCCCCC REV:CTTCTTGAGGGACAGCAGGAC |
| ***Ecscr*** | FWD:CAGGTATTGGGAAGATGGGC REV: TGTGGGAGTTGATGGGCA |
| ***Emb*** | FWD: CCCCGGTACAGAAAAACTGACT REV: GCACGTTGAGGGCATCTTTG |
| ***Emp1*** | FWD: AAGAGAGGACCAGACCAGCA REV: ATCCAGACGTTGGCAATGGT |
| ***Fam26f*** | FWD: GGCTCAGTCTCAGGTGTTCG REV: TCGCATTCTCTGCAGCTTGA |
| ***Fat3*** | FWD: GTTTGACATCGTCGGGGG REV:TTGTTCCATCGGTGACCTCG |
| ***Gpr132*** | FWD: TCAGGACTGGCTTGGGTCATT REV: GAGGTGCTCTGAAGAACGGA |
| ***Gpr141*** | FWD: GAGTCTGAGCATGGACAAAGGA  REV: CGTAGTAAGACTGATTCGTTCCA |
| ***Gpr165*** | FWD: GCCATCATAGGCAACTGGGT  REV: TAGCGCACCATAGTGAAGGG |
| ***Ifitm6*** | FWD: CATCCACTGTGGTTTGCATCA REV: GTCCCTGGACTTCACCGAGTAG |
| ***Iqgap2*** | FWD:AGGTACAACTCAATATTCAGGACTT  REV: GGCACGTCACTTTCCGTAGA |
| ***Jam2*** | FWd: CTCTGCTTCCACCTCTCAGGG REV:CTTATGATAGTCCAGGGCGACGAT |
| ***Kcnd1*** | FWD: TGGTACCACCAATGCCTTCC REV: CGTGACACGTAGTCTTCTCCA |
| ***Kcnn4*** | FWD: TGCCCTGGAGAAGAGAATCG REV: CTTCTGTGAGTTCATGTGGAGC |
| ***Lilr4b*** | FWD: GTGCTGCTATACCTTGCTCT REV: CAGGAACCCCAACACCAGAT |
| ***Lilra6*** | FWD: GTCTCAGCCACAGAGAACCA REV: TGCAGGGTGGGTCTGTCTAT |
| ***Mrc2*** | FWD: ACTTCATCTCTTGGGCACCG REV: CCAGTCCTCTTGTCTGGCTG |
| ***Ms4a6d*** | FWD: TTTTGGCATCTGTTCCCTCCA REV: CTAGGCTGCTGTGAACCAAAGG |
| ***Ms4a7*** | FWD: GGGCAGTTCTGGAAAGAGACA REV: TGCCAGGGATGCAAATGAACC |
| ***Ms4a8a*** | FWD: CAGGATCTCTCTCAGTGGCG REV: GCCTGTTCTCACACCCAAGTT |
| ***Msr1*** | FWD:CGGATCAAGATCAGTATAACTCCCT REV: ATGGCATGACACAGGAACCA |
| ***P2ry12*** | FWD: CAAGGGGTGGCATCTACCTG REV: AGCCTTGAGTGTTTCTGTAGGG |
| ***Pdpn*** | FWD: GCAGGGGATGAAACGCAGA REV: TAGCTCTTTAGGGCGAGAACC |
| ***Plxna4*** | FWD: GTGCAAGGAAGCTTTTGCCG REV: ACAGCACCCGCATGGTATAG |
| ***Pros1*** | FWD:CAGCAATGAGGGTCCTGAGC REV:ACGCTCTTTGGACAAGAAGTTTG |
| ***Ptprm*** | FWD: CTTAGGCTCGGGTCTCCCTT REV: AGGCAGCCACCTGAAAATGT |
| ***Rtn4rl1*** | FWD: CCGGGTTTGGGCTCACC REV:ACACAGCACCCTTTGCG |
| ***Scamp5*** | FWD: CAGGTATTGGGAAGATGGGC REV: GGTAGTAGAGGCGCTTGGTC |
| ***Scarf1*** | FWD: AGTGCAGTCGTAAGTGCCAG REV: GGCCACAGCTTTCTTTGCAC |
| ***Sdk1*** | FWD: TCCTTTCCATCATAGAGCCACC REV: GGAGTTTGCTGAGGGGAACA |
| ***Sema4g*** | FWD:TGGCTGAGATTCAGGCTGTC REV:GAATCTGTGATGCACGAGCC |
| ***Sgce*** | FWD: CACAGCAGAACTTGCCACAC REV: TGGAAGGGAAAGGGGTGAGA |
| ***Sirpb1a*** | FWD: TCTCCTAGATGCCTGGACCC REV: CTCTCTCACAGCTGCTCCTTT |
| ***Slc12a2*** | FWD: GGAGTTGTGGTTATCCGCCT REV:CCATCCTCTTCCTCATCTTTCTGT |
| ***Slc46a1*** | FWD: CGCTCACCACACAGTACCTT REV: GTCAGGGTCTCCACTTCCTTC |
| ***Slco2b1*** | FWD: GACTGGGGGCCACAATTCTC REV:GGACAAAGAACTTGATGTTGTGGA |
| ***Slco3a1*** | FWD: TTTCTGGGTGGCCTTTTGGT REV: CACGTGCTGAGTTGTTTCCG |
| ***Slfn4*** | FWD: GCCAGAAAGATGCTTCTGTATCC REV: GTCCTTACTCTGCCCCGAAC |
| ***Smo*** | FWD: GAGCCTTTGCGCTACAACG REV: CGGAGGCCGGACCAGA |
| ***Tmem204*** | FWD: GGTAAGGGAGTCACAAGCCC  REV: GGTAAGGGAGTCACAAGCCC |
| ***Trem1*** | FWD: GCGTGTTCTTTGTCTCAGAAGTC REV: CTGGTAGTCTCTGCCAAGCC |
| ***Wfdc17*** | FWD: GTGTCTTGCCCCAAAGAATTT REV: TTTGCAGACATGACCACAGC |
